# Appendicular lean mass and the risk of stroke and Alzheimer’s disease: a mendelian randomization study

**DOI:** 10.1186/s12877-024-05039-5

**Published:** 2024-05-18

**Authors:** Yueli Zhu, Feng Zhu, Xiaoming Guo, Shunmei Huang, Yunmei Yang, Qin Zhang

**Affiliations:** 1 Department of Geriatrics, The First Affiliated Hospital, School of Medicine, Zhejiang University, Hangzhou, China; 2 Key Laboratory of Diagnosis and Treatment of Aging and Physic-chemical Injury Diseases of Zhejiang Province, The First Affiliated Hospital, School of Medicine, Zhejiang University, Hangzhou, Zhejiang China; 3 Department of Neurosurgery, The Second Affiliated Hospital, School of Medicine, Zhejiang University, Hangzhou, China

**Keywords:** Appendicular lean mass, Sarcopenia, Stroke, Alzheimer’s disease, Mendelian randomization

## Abstract

**Background:**

Appendicular lean mass (ALM) is a good predictive biomarker for sarcopenia. And previous studies have reported the association between ALM and stroke or Alzheimer’s disease (AD), however, the causal relationship is still unclear, The purpose of this study was to evaluate whether genetically predicted ALM is causally associated with the risk of stroke and AD by performing Mendelian randomization (MR) analyses.

**Methods:**

A two-sample MR study was designed. Genetic variants associated with the ALM were obtained from a large genome-wide association study (GWAS) and utilized as instrumental variables (IVs). Summary-level data for stroke and AD were generated from the corresponding GWASs. We used random-effect inverse-variance weighted (IVW) as the main method for estimating causal effects, complemented by several sensitivity analyses, including the weighted median, MR-Egger, and MR-pleiotropy residual sum and outlier (MR-PRESSO) methods. Multivariable analysis was further conducted to adjust for confounding factors, including body mass index (BMI), type 2 diabetes mellitus (T2DM), low density lipoprotein-C (LDL-C), and atrial fibrillation (AF).

**Results:**

The present MR study indicated significant inverse associations of genetically predicted ALM with any ischemic stroke ([AIS], odds ratio [OR], 0.93; 95% confidence interval [CI], 0.89–0.97; *P* = 0.002) and AD (OR, 090; 95% CI 0.85–0.96; *P* = 0.001). Regarding the subtypes of AIS, genetically predicted ALM was related to the risk of large artery stroke ([LAS], OR, 0.86; 95% CI 0.77–0.95; *P* = 0.005) and small vessel stroke ([SVS], OR, 0.80; 95% CI 0.73–0.89; *P* < 0.001). Regarding multivariable MR analysis, ALM retained the stable effect on AIS when adjusting for BMI, LDL-C, and AF, while a suggestive association was observed after adjusting for T2DM. And the estimated effect of ALM on LAS was significant after adjustment for BMI and AF, while a suggestive association was found after adjusting for T2DM and LDL-C. Besides, the estimated effects of ALM were still significant on SVS and AD after adjustment for BMI, T2DM, LDL-C, and AF.

**Conclusions:**

The two-sample MR analysis indicated that genetically predicted ALM was negatively related to AIS and AD. And the subgroup analysis of AIS revealed a negative causal effect of genetically predicted ALM on LAS or SVS. Future studies are required to further investigate the underlying mechanisms.

**Supplementary Information:**

The online version contains supplementary material available at 10.1186/s12877-024-05039-5.

## Introduction

Sarcopenia, which is characterized by loss of skeletal muscle mass and strength, is a geriatric syndrome and has been reported to be related to increased risk of many adverse outcomes, including physical disability, poor quality of life and even death [1, 2]. And it is of great value to investigate the potential linkage between sarcopenia and aging-related diseases, which will contribute to the early diagnosis and timely interventions.

Stroke is now becoming a leading cause of mortality and disability, especially in low- and middle-income countries [3]. It has have revealed that prestroke sarcopenia can affect stroke severity in elderly patients [4]. In addition, prestroke sarcopenia was an independent predictor for poorer functional outcome at 3 months after acute stroke [5].

As for another aging-related disease, Alzheimer’s disease (AD) is the most prevalent neurodegenerative disease and the major cause of dementia. The close relationship between sarcopenia and cognitive impairment has been observed [6, 7]. And the prevalence of cognitive impairment was 40% in patients with sarcopenia [6].

However, the causal effects of sarcopenia on stroke and AD still remain unclear, as it will be very challenging based on the inherent risk of bias due to confounding or reverse causality in the observational studies. Appendicular lean mass (ALM) is the sum of lean mass for both arms and legs and can be regarded as a major index to define sarcopenia [8]. Recently, a genome-wide association study (GWAS) identified ALM-associated single-nucleotide polymorphisms (SNPs) [9], which provided an opportunity to explore the causal associations of ALM with the risk of stroke and AD by performing Mendelian randomization (MR) analyses.

MR is a powerful approach for evaluating the causal links between clinical exposures and outcomes [10]. Genetic variants associated with the exposures are employed as instrumental variables (IVs) [10]. Since alleles are randomly assigned to the offspring and can remain constant after conception, the MR approach can avoid some limitations of conventional observational studies and reduce the influence of unmeasured confounding and reverse causality. Hence, in the present study, we aimed to use the two-sample MR analysis to elucidate the causal relationships between genetically predicted ALM and the risk of stroke subtypes (including large artery stroke [LAS], small vessel stroke [SVS], and cardioembolic stroke [CES]) as well as AD.

## Methods

### Study design

**Fig. 1:**
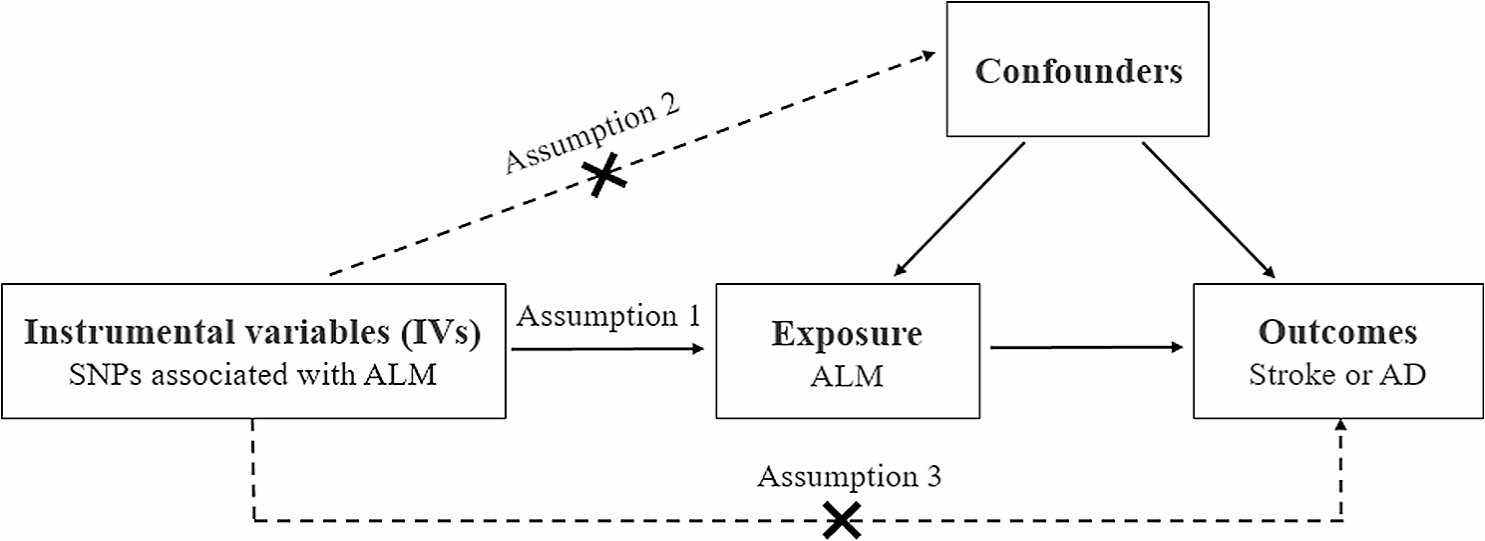
Schematic representation of Mendelian randomization analysis. SNP, single-nucleotide polymorphism; ALM, appendicular lean mass; AD, Alzheimer’s disease

A two-sample MR was performed to evaluate the causal effects of ALM on the risk of stroke and AD (Fig. 1). The present MR study is based on three predominant assumptions [11]. First, selected SNPs are associated with ALM; second, SNPs are not associated with other confounders; third, SNPs affect the risk of stroke and AD only through ALM, but not other pathways.

### Ethics approval

All analyses of this study were based on the publicly available data, and ethical approval had been obtained in the original studies.

### Selection of IVs for ALM

We used a GWAS of ALM to identify independent SNPs which were significantly associated with ALM from the UK Biobank with 450,243 European ancestry participants (Table 1) [9]. In this GWAS, ALM was measured using bioelectrical impedance analysis (BIA) for the sum of fat-free mass at the arms and legs [9]. The total 1059 SNPs associated with ALM (*P* < 5.0 × 10^− 9^) were obtained for the analyses, which explained 15.5% of the phenotypic variance. The F statistic was used to evaluate the weak instrument bias of each SNP using the formula equation: F = R^2^ × (*N* − 2) / (1 − R^2^), where R^2^ shows the proportion of variance of ALM and N represents the sample size [12]. R^2^ of each SNP was calculated by using the formula R^2^ = 2 × effect allele frequency × (1 − effect allele frequency) ×Beta^2^. F statistic > 10 indicated that the selected SNP can be recommended as an indication of strong IV. These ALM-associated SNPs were further tested whether there was a linkage disequilibrium. Finally, 810 of these SNPs passed the selection criteria and were included for further MR analysis (r^2^ < 0.1; region size, 3000 kb). Proxy SNPs in linkage disequilibrium (r^2^ > 0.8) were searched online (http://snipa.helmholtz-muenchen.de/snipa3/) and used if the ALM-associated SNPs were not available in the datasets of stroke or AD (Supplementary Table 1).

**Table 1:** Details of data sources involved in the present MR study

Phenotype	Consortium	Ancestry	Sample size	Cases	Use in this MR
ALM	UKB^[1]^	European	450,243	/	Exposure
AIS	MEGASTROKE^[2]^	European	440,328	34,217	Outcome
LAS			410,484	4,373	Outcome
SVS			411,497	5,386	Outcome
CES			413,304	7,193	Outcome
AD	IGAP^[3]^	European	63,926	21,982	Outcome
BMI	GERA, GIANT^[4]^	94.1% European	458,721	/	Confounder
T2DM	DIAGRAM, GERA, UKB^[5]^	99.4% European	659,316	62,892	Confounder
LDL-C	GERA^[6]^	80.9% European	94,674	/	Confounder
AF	AFHRC^[7]^	84.2% European	588,190	65,446	Confounder

ALM, appendicular lean mass; AIS, any ischemic stroke; LAS, large artery stroke; SVS, small vessel stroke; CES, cardioembolic stroke; AD, Alzheimer’s disease; BMI, body mass index; T2DM, type 2 diabetes mellitus; LDL-C, low density lipoprotein-C; AF, atrial fibrillation; UKB, UK Biobank; IGAP, International Genomics of Alzheimer’s Project; GERA, Genetic Epidemiology Research on Aging; GIANT, Genetic Investigation of ANthropometric Traits; DIAGRAM, DIAbetes Genetics Replication and Meta-analysis; AFHRC, Atrial Fibrillation Haplotype Reference Consortium

**Reference**

[1] Pei YF, Liu YZ, Yang XL, Zhang H, Feng GJ, Wei XT, et al. The genetic architecture of appendicular lean mass characterized by association analysis in the UK Biobank study. Commun Biol. 2020;3(1):608. doi: 10.1038/s42003-020-01334-0

[2] Malik R, Chauhan G, Traylor M, Sargurupremraj M, Okada Y, Mishra A, et al. Multiancestry genome-wide association study of 520,000 subjects identifies 32 loci associated with stroke and stroke subtypes. Nat Genet. 2018;50(4):524–537. doi: 10.1038/s41588-018-0058-3

[3] Kunkle BW, Grenier-Boley B, Sims R, Bis JC, Damotte V, Naj AC, et al. Genetic meta-analysis of diagnosed Alzheimer’s disease identifies new risk loci and implicates Aβ, tau, immunity and lipid processing. Nat Genet. 2019;51(3):414–430. doi: 10.1038/s41588-019-0358-2

[4] Hoffmann TJ, Choquet H, Yin J, Banda Y, Kvale MN, Glymour M, et al. A Large Multiethnic Genome-Wide Association Study of Adult Body Mass Index Identifies Novel Loci. Genetics. 2018;210(2):499–515. doi: 10.1534/genetics.118.301479

[5] Xue A, Wu Y, Zhu Z, Zhang F, Kemper KE, Zheng Z, et al. Genome-wide association analyses identify 143 risk variants and putative regulatory mechanisms for type 2 diabetes. Nat Commun. 2018;9(1):2941. doi: 10.1038/s41467-018-04951-w

[6] Hoffmann TJ, Theusch E, Haldar T, Ranatunga DK, Jorgenson E, Medina MW, et al. A large electronic-health-record-based genome-wide study of serum lipids. Nat Genet. 2018 Mar;50(3):401–413. doi: 10.1038/s41588-018-0064-5

[7] Roselli C, Chaffin MD, Weng LC, Aeschbacher S, Ahlberg G, Albert CM, et al. Multi-ethnic genome-wide association study for atrial fibrillation. Nat Genet. 2018;50(9):1225–1233. doi: 10.1038/s41588-018-0133-9

### Outcomes data sources

Summary statistics for the association between the ALM-related genetic variants and stroke were extracted from the MEGASTROKE consortium, which included 34,217 ischemic stroke cases and 406, 111 controls with European ancestry (Table 1) [13]. In this GWAS study, 34,217 ischemic cases were further classified as LAS (*n* = 4373), SVS (*n* = 5386), and CES (*n* = 7193) according to the Trial of ORG 10,172 in Acute Stroke Treatment (TOAST) criteria [13]. Genetic variants were measured and imputed in dosage format using an additive genetic model with a minimum of sex and age as covariates [13].

Summary statistics for the association between the ALM-related genetic variants and AD were obtained from a GWAS meta-analysis of International Genomics of Alzheimer’s Project (IGAP) stage 1 discovery study with 21,982 cases and 41,944 cognitively normal controls with European ancestry (Table 1) [14]. And all these stage 1 samples were from the following four consortia: Alzheimer Disease Genetics Consortium (ADGC; consisting of 14,428 cases and 14,562 controls), Cohorts for Heart and Aging Research in Genomic Epidemiology (CHARGE; consisting of 2137 cases and 13,474 controls) consortium, The European Alzheimer’s Disease Initiative (EADI; consisting of 2240 cases and 6631 controls), and Genetic and Environmental Risk in AD/Defining Genetic, Polygenic and Environmental Risk for Alzheimer’s Disease Consortium (GERAD/PERADES; consisting of 3177 cases and 7277 controls). Age, sex, and principal components were used as covariates in the analysis [14].

### Statistical analysis

We conducted the two-sample MR analyses to assess the causal associations of ALM with the risk of stroke and AD. In the main analyses, we used the random effects inverse-variance weighted (IVW) approach to estimate the causal effects. Besides, sensitivity analyses were performed to assess the robustness of the IVW results by using the weighted median, MR-Egger, and MR-pleiotropy residual sum and outlier (MR-PRESSO) methods. The weighted median method can provide valid estimates as long as at least 50% of the information in the analysis comes from valid IVs [15]. MR-Egger method was conducted to assess and adjust for the bias due to directional pleiotropy [16]. The MR-PRESSO method was used to detect outlying SNPs which are potentially horizontally pleiotropic and assess whether exclusion of these outlying SNPs influences the causal estimates [17]. Cochran’s Q statistic was utilized to assess the heterogeneity among SNPs. Heterogeneity was considered to exist if the *P* value of Cochran’s Q statistic was less than 0.05, and then random effects IVW approaches were used. The web-based application was used to calculate the statistical power (http://cnsgenomics.com/shiny/mRnd/).

Besides, multivariable MR analysis was conducted for the purpose of adjustment for confounders [18]. The following four covariates were taken into account in the multivariable analysis, including body mass index (BMI), type 2 diabetes mellitus (T2DM), low density lipoprotein-C (LDL-C), and atrial fibrillation (AF). We used publicly available summary statistics for BMI from Hoffmann et al. [19], T2DM from Xue et al. [20], LDL-C from Hoffmann et al. [21], and AF from the Haplotype Reference Consortium (Table 1) [22]. The Bonferroni-corrected significance threshold was set to *P* < 0.01 (corrected *P* value 0.05 / 5 outcomes). And a *P* value between 0.01 and 0.05 was defined as a suggestive association between exposure and outcome. All analyses were conducted using the TwoSampleMR [23], MendelianRandomization [24], and MR-PRESSO packages [17] in R software (Version 4.1.3).

## Results

### Influence of genetically predicted ALM on the risk of stroke

There was moderate heterogeneity (*P* for Cochran’s Q < 0.05) in the estimated effects of ALM on stroke and AD, but without pleiotropies (*P* for intercept > 0.05) (Supplementary Table 2). Therefore, the multiplicative random effects IVW method was applied for more reliable estimates.

**Fig. 2:**
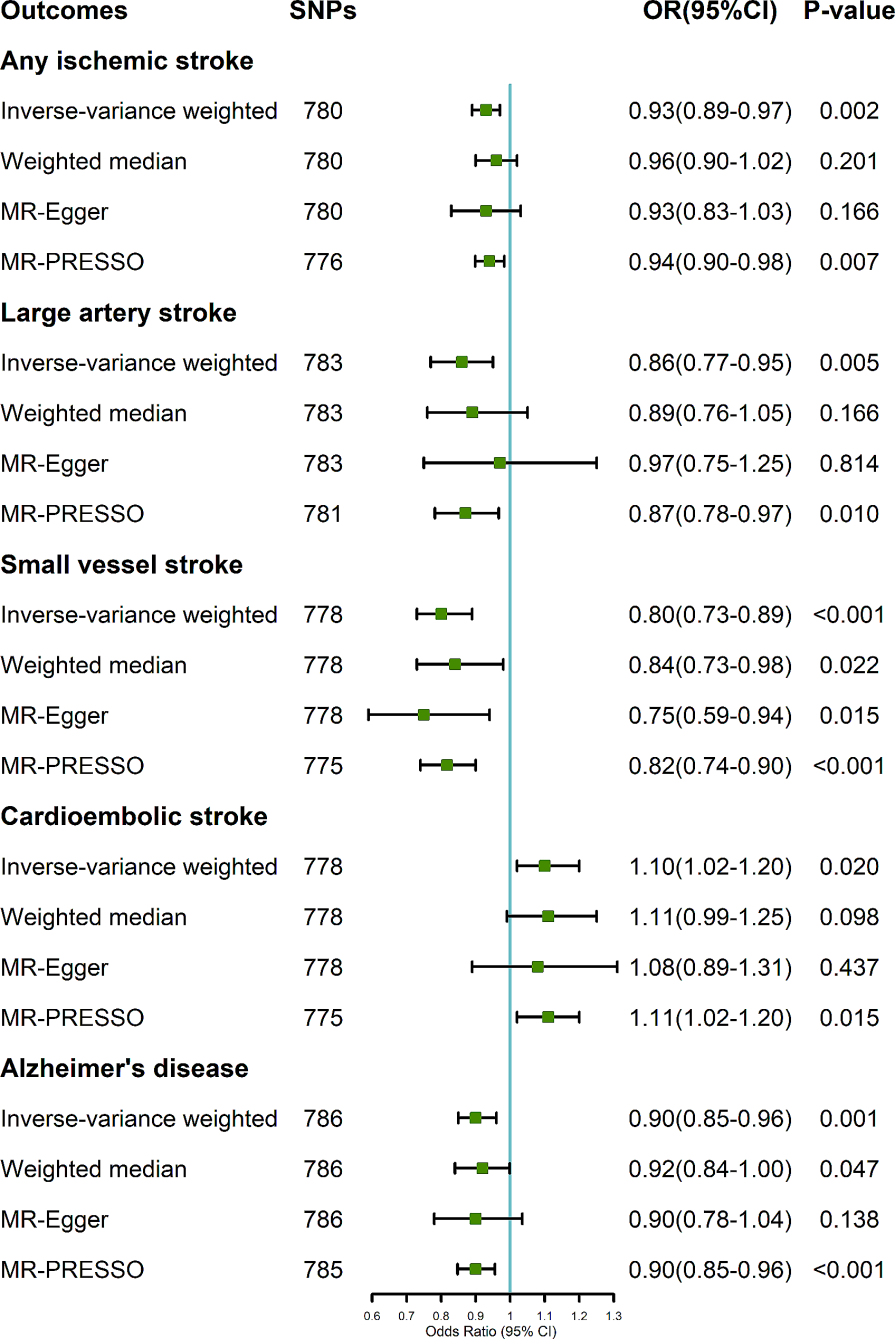
Causal effect estimates of genetically predicted ALM on stroke and AD. *MR-PRESSO outlier detected: rs4858605, rs42039, rs3184504, rs118127175 (for AIS); rs3184504, rs732716 (for LAS); rs72938315, rs10824747, rs3184504 (for SVS); rs295139, rs7633464, rs10993370 (for CES); rs4663096 (for AD). AIS, any ischemic stroke; LAS, large artery stroke; SVS, small vessel stroke; CES, cardioembolic stroke; AD, Alzheimer’s disease; SNP, single nucleotide polymorphism; OR, odds ratio; CI, confidence interval

The overall IVW MR analyses revealed a negative relationship between genetically predicted ALM and the risk of any ischemic stroke ([AIS], odds ratio [OR], 0.93; 95% confidence interval [CI], 0.89–0.97; *P* = 0.002; Fig. 2). Subgroup analysis of AIS showed that genetically predicted ALM was associated with the risk of LAS(OR, 0.86; 95% CI 0.77–0.95; *P* = 0.005) and SVS (OR, 0.80; 95% CI 0.73–0.89; *P* < 0.001).

As for sensitivity analyses, we found a significant causal association between ALM and the risk of AIS using MR-PRESSO method after excluding four potential outliers (*P* = 0.007; Fig. 2). The suggestive causal association was observed between genetically predicted ALM and LAS using MR-PRESSO method after excluding two potential outliers (*P* = 0.010). Besides, genetically predicted ALM was suggestively associated with the risk of SVS using weighted median and MR-Egger methods (both *P* < 0.05), while the causal significant relationship was found using MR-PRESSO method after excluding three potential outliers (*P* < 0.001).

### Influence of genetically predicted ALM on the risk of AD

The overall IVW MR analyses indicated a causal effect of genetically predicted ALM on the risk of AD (OR, 0.90; 95% CI 0.85–0.96; *P* = 0.001; Fig. 2).

In the sensitivity analysis, the significant causal association was found between genetically predicted ALM and AD using MR-PRESSO method after excluding one potential outliers (*P* < 0.001), while genetically predicted ALM was suggestively associated with the risk of AD using weighted median method (*P* = 0.047).

### Multivariable MR analysis

To further investigate the causal associations of genetically predicted ALM with the risk of stroke and AD, multivariable MR analyses were performed including BMI, T2DM, LDL-C, and AF.

**Fig. 3:**
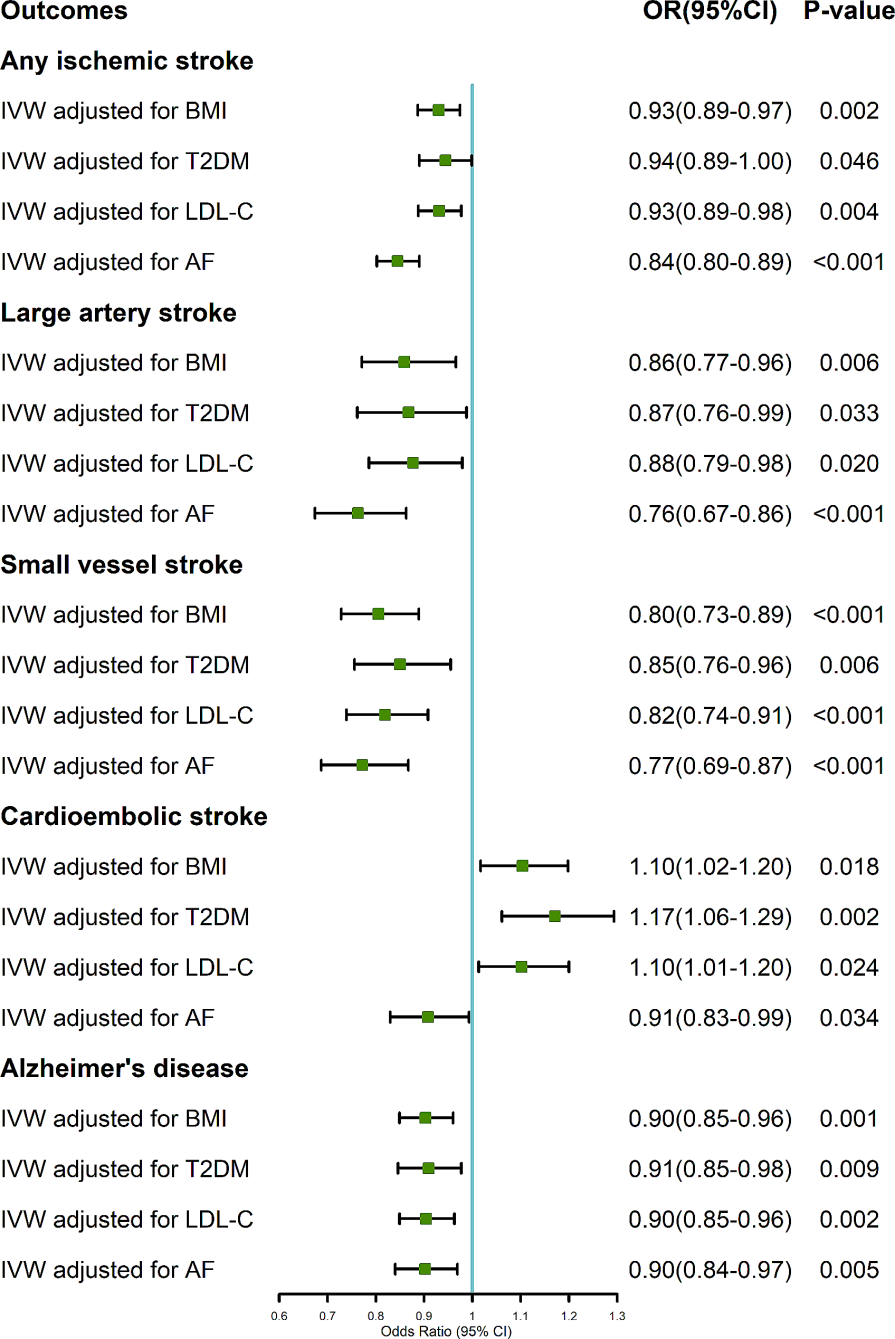
Multivariable Mendelian randomization analysis of the causal associations of genetically predicted ALM with the risk of stroke and AD. AIS, any ischemic stroke; LAS, large artery stroke; SVS, small vessel stroke; CES, cardioembolic stroke; AD, Alzheimer’s disease; IVW, inverse-variance weighted; BMI, body mass index; T2DM, type 2 diabetes mellitus; LDL-C, low density lipoprotein-C; AF, atrial fibrillation; OR, odds ratio; CI, confidence interval

The multivariable MR analysis revealed that genetically predicted ALM retained the stable effect on AIS when adjusting for BMI (OR, 0.93; 95% CI 0.89–0.97; *P* = 0.002; Fig. 3), LDL-C (OR, 0.93; 95% CI 0.89–0.98; *P* = 0.004), and AF (OR, 0.84; 95% CI 0.80–0.89; *P* < 0.001), while a suggestive association was observed after adjusting for T2DM (OR, 0.94; 95% CI 0.89-1.00; *P* = 0.046). Regarding LAS, the estimated effect of ALM was significant after adjustment for BMI (OR, 0.86; 95% CI 0.77–0.96; *P* = 0.006) and AF (OR, 0.76; 95% CI 0.67–0.86; *P* < 0.001), while a suggestive association was found after adjustment for T2DM (OR, 0.87; 95% CI 0.76–0.99; *P* = 0.033) and LDL-C (OR, 0.88; 95% CI 0.79–0.98; *P* = 0.020). The estimated effects of ALM on SVS and AD were unchanged after adjustment for BMI (OR, 0.80; 95% CI 0.73–0.89; *P* < 0.001 for SVS; OR, 0.90; 95% CI 0.85–0.96; *P* = 0.001 for AD ), T2DM (OR, 0.85; 95% CI 0.76–0.96; *P* = 0.006 for SVS; OR, 0.91; 95% CI 0.85–0.98; *P* = 0.009 for AD), LDL-C (OR, 0.82; 95% CI 0.74–0.91; *P* < 0.001 for SVS; OR, 0.90; 95% CI 0.85–0.96; *P* = 0.002 for AD), and AF (OR, 0.77; 95% CI 0.69–0.87; *P* < 0.001 for SVS; OR, 0.90; 95% CI 0.84–0.97; *P* = 0.005 for AD). Intriguingly, the association between ALM and CES was directionally inconsistent with the IVW MR analysis after adjustment for AF, which revealed a suggestive negative relationship (OR, 0.91; 95% CI 0.83–0.99; *P* = 0.034).

## Discussion

In the present study, we conducted a two-sample MR study to investigate whether genetically predicted ALM was causally associated with the risk of stroke and AD. Our findings showed the significant negative relationship between genetically predicted ALM and the risk of AIS, LAS, SVS, and AD. Multivariable MR analysis suggested that ALM retained the stable effect on AIS when adjusting for BMI, LDL-C, and AF, while a suggestive association was observed after adjusting for T2DM. And the estimated effect of ALM on LAS was significant after adjustment for BMI and AF, while a suggestive association was found after adjusting for T2DM and LDL-C. Besides, the estimated effects of ALM were still significant on SVS and AD after adjustment for BMI, T2DM, LDL-C, and AF.

ALM is mainly determined by skeletal muscle and has a good predictive power for sarcopenia, which is mainly due to the progressive loss of skeletal muscle mass and strength [2, 9]. In addition, ALM is highly heritable and can be a suitable trait for sarcopenia-related genetic analyses [25].

Ischemic stroke is one of the leading causes of mortality and long-term disability worldwide. It has been reported that sarcopenia was related to elevated prevalence of stroke in South korean men aged ≥ 50 years [26]. Besides, increased skeletal muscle mass may contribute to protect against silent infarction [27]. However, the relationship between genetically predicted ALM and stroke has not been explored yet. In this MR study, we found significant negative associations between ALM and the risk of AIS, LAS, and SVS. It may be attributed to chronic low-grade inflammation, which can promote the loss of muscle mass, strength, and function on account of the influences on both muscle protein breakdown and synthesis [28]. What’s more, inflammation can mediate aberrant platelet aggregation, which can stick to the surface of endothelial cells and induce local ischemia and hypoxia, even resulting in tissue death. Thus, individuals with signs of inflammation or corresponding biomarkers are considered to have an elevated risk of stroke [29]. In addition, it has been reported that there is an inverse association between peripheral lean mass and endothelial dysfunction, suggesting that low ALM may play an important role in the decline of endothelial function [30]. As we know, endothelial cells play an important role in maintaining vascular homeostasis. And vascular endothelial dysfunction is critically related to the development of cardiovascular diseases, including stroke. Therefore, chronic inflammation and vascular endothelial dysfunction are possible factors associating ALM and stroke.

And our present MR study showed a significant causal association between genetically predicted ALM and the risk of AD. As we know, it has been reported that there was an inverse relationship between lean mass and AD incidence [31, 32]. And this relationship may be explained by several mechanisms. Chronic inflammation and oxidative stress have been proven to mediate low lean mass and AD in the elderly [33]. Besides, low muscle mass but not muscle strength, has been found to be independently related to parietal gray matter volume atrophy in middle-aged adults [34]. And the parietal lobe is involved in the early stage of AD [35], suggesting that parietal lobe involvement might lead to cognitive impairment in individuals with low muscle mass. Finally, serum brain-derived neurotrophic factor (BDNF) had a positive correlation with muscle mass [36]. And the decreased level of BDNF can lead to cognitive deterioration, while greater levels of BDNF by exercise training can increase hippocampal volume and improve cognitive function [37].

Therefore, this study provided reliable causal evidence for the protective effects of ALM on the risk of stroke and AD. Recently, a randomized controlled trial has explored a plausible multicomponent intervention based on physical activity with technological support and nutritional counselling for sarcopenia [38]. Our findings inform the development of physical interventions targeting low ALM to reduce the risk of stroke and AD.

There are several strengths in this study. One strength of this study is the MR design. We used the MR method to investigate the causal association of genetically predicted ALM with the risk of stroke and AD based on ALM-related SNPs and effects of SNPs on the outcomes from GWASs, which can reduce bias induced by residual confounding and reverse causality. Second, sensitivity analyses were applied to evaluate the robustness of our study. Third, some potential confounding factors were further analyzed by multivariable MR methods, including BMI, T2DM, LDL-C, and AF.

However, several limitations in this study should be considered. First, this study utilized ALM data from the UK Biobank (UKB), which was measured using BIA rather than DXA. As we know, BIA is an indirect measurement method to measure muscle mass and may be less accurate than DXA, which could affect the results. Second, pleiotropy, especially the horizontal pleiotropy, is generally inevitable in MR analysis which would be likely to affect the reliability of our results, despite the lack of evidence from MR-Egger and MR-PRESSO methods. Besides, multivariable MR analyses were further applied by adjusting for some confounders. However, the pleiotropy could not be fully ruled out in the MR analysis. Third, the GWAS data was mainly derived from European, and caution should be exercised when generalizing our findings to different populations, particularly those of non-European ancestry. Fourth, we used summary statistics in this study and had no access to the patient-level data. Given the different incidences of low ALM, stroke, and AD by age and sex, we believe that investigating the casual associations of ALM with the risk of stroke and AD based on different ages and sexes would be of value.

## Conclusions

In conclusion, our two-sample MR analysis provided genetic support for the negative causal effects of genetically predicted ALM on the risk of AIS, LAS, SVS, and AD. Future studies are required to further confirm our findings and investigate the underlying mechanisms.

### Electronic supplementary material

Below is the link to the electronic supplementary material.

Supplementary Material 1

## Data Availability

The data used in this study was obtained from public databases and previous studies. Further information is available from the corresponding author upon request.

## Abbreviations

AD: Alzheimer’s disease
ADGC: Alzheimer Disease Genetics Consortium
AF: Atrial fibrillation
AIS: Any ischemic stroke
ALM: Appendicular lean mass
BDNF: Brain-derived neurotrophic factor
BIA: Bioelectrical impedance analysis
BMI: Body mass index
CES: Cardioembolic stroke
CHARGE: Cohorts for Heart and Aging Research in Genomic Epidemiology
EADI: European Alzheimer’s Disease Initiative
GERAD/PERADES: Genetic and Environmental Risk in AD/Defining Genetic, Polygenic and Environmental Risk for Alzheimer’s Disease Consortium
GWAS: Genome-wide association study
IGAP: International Genomics of Alzheimer’s Project
IVs: Instrumental variables
IVW: Inverse-variance weighted
LAS: Large artery stroke
LDL-C: Low density lipoprotein-C
MR: Mendelian randomization
MR-PRESSO: MR-pleiotropy residual sum and outlier
SNPs: Single-nucleotide polymorphisms
SVS: Small vessel stroke
T2DM: Type 2 diabetes mellitus
TOAST: Trial of ORG 10172 in Acute Stroke Treatment
