# Supplementary material for: Appendicular lean mass and the risk of stroke and Alzheimer’s disease: a mendelian randomization study
Source: BMC Geriatr. 2024 May 18;24:438. doi: 10.1186/s12877-024-05039-5 (PMC11102192; doi:10.1186/s12877-024-05039-5)
Supplement: Supplementary file 1 — Supplementary Material 1 [file 12877_2024_5039_MOESM1_ESM.docx]

**Supplementary Table 1**. Characteristics of the genetic variants significantly associated with ALM.

|  | SNP | EA | OA | EAF | Beta | SE | P | F-statistics | AIS proxy, excluded or outliers | LAS proxy, excluded or outliers | SVS proxy excluded or outliers | CES proxy excluded or outliers | AD proxy, excluded or outliers |
| --- | --- | --- | --- | --- | --- | --- | --- | --- | --- | --- | --- | --- | --- |
| 1 | rs2294487 | a | g | 0.4105 | 0.02 | 0.002 | 5.51E-14 | 87.2 |  |  |  |  |  |
| 2 | rs377599 | t | c | 0.383 | 0.02 | 0.002 | 3.50E-29 | 85.1 |  |  |  |  |  |
| 3 | rs2232460 | a | g | 0.3343 | 0.01 | 0.002 | 2.79E-13 | 20.0 |  |  |  |  |  |
| 4 | rs11122154 | a | t | 0.1665 | 0.02 | 0.003 | 3.10E-15 | 50.0 |  |  |  |  |  |
| 5 | rs301804 | c | g | 0.6867 | 0.02 | 0.002 | 4.05E-17 | 77.5 |  |  |  |  |  |
| 6 | rs3903151 | a | g | 0.5794 | -0.02 | 0.002 | 1.34E-16 | 87.8 |  |  |  |  |  |
| 7 | rs11121615 | t | c | 0.6894 | -0.02 | 0.002 | 1.83E-22 | 77.1 |  |  |  |  |  |
| 8 | rs7538833 | t | c | 0.3761 | 0.01 | 0.002 | 4.64E-10 | 21.1 |  |  |  |  |  |
| 9 | rs2284747 | t | c | 0.5213 | 0.02 | 0.002 | 2.34E-16 | 89.9 |  |  |  |  |  |
| 10 | rs1472565 | t | c | 0.5333 | 0.01 | 0.002 | 8.56E-13 | 22.4 |  |  |  |  |  |
| 11 | rs212526 | t | c | 0.3993 | -0.02 | 0.002 | 3.84E-29 | 86.4 |  |  |  |  |  |
| 12 | rs7543136 | t | c | 0.7209 | -0.02 | 0.002 | 9.96E-24 | 72.5 |  |  |  |  |  |
| 13 | rs6682646 | a | g | 0.1721 | -0.02 | 0.002 | 4.16E-12 | 51.3 |  |  |  |  |  |
| 14 | rs4274112 | a | g | 0.6267 | 0.02 | 0.002 | 2.47E-28 | 84.3 |  |  |  |  |  |
| 15 | rs6687475 | c | g | 0.7436 | -0.01 | 0.002 | 5.61E-12 | 17.2 |  |  |  |  |  |
| 16 | rs591833 | a | g | 0.1334 | 0.03 | 0.003 | 3.55E-21 | 93.7 |  |  |  |  |  |
| 17 | rs182524742 | t | c | 0.011 | -0.06 | 0.009 | 2.92E-10 | 35.3 |  |  |  | Excluded |  |
| 18 | rs12116935 | a | g | 0.614 | 0.02 | 0.002 | 2.46E-23 | 85.4 |  |  |  |  |  |
| 19 | rs4360494 | c | g | 0.5541 | -0.02 | 0.002 | 7.88E-26 | 89.0 |  |  |  |  |  |
| 20 | rs61781391 | a | g | 0.2172 | 0.02 | 0.002 | 1.16E-11 | 61.2 |  |  |  |  |  |
| 21 | rs3737815 | a | c | 0.1684 | -0.02 | 0.003 | 1.70E-09 | 50.4 |  |  |  |  |  |
| 22 | rs2885697 | t | g | 0.665 | -0.03 | 0.002 | 9.21E-60 | 180.6 |  |  |  |  |  |
| 23 | rs1084086 | t | g | 0.2967 | 0.01 | 0.002 | 9.72E-13 | 18.8 |  |  |  |  |  |
| 24 | rs12074850 | a | g | 0.9102 | -0.04 | 0.003 | 2.72E-33 | 117.8 |  |  |  |  |  |
| 25 | rs142478128 | t | c | 0.9713 | -0.04 | 0.006 | 1.67E-10 | 40.2 |  |  | Excluded |  | Excluded |
| 26 | rs80206427 | t | c | 0.965 | -0.04 | 0.005 | 1.87E-19 | 48.7 |  |  |  |  |  |
| 27 | rs670318 | t | c | 0.0485 | -0.04 | 0.004 | 2.52E-21 | 66.5 |  |  |  |  |  |
| 28 | rs2025609 | c | g | 0.1489 | -0.02 | 0.003 | 2.00E-12 | 45.7 |  |  |  |  |  |
| 29 | rs34517439 | a | c | 0.1221 | 0.04 | 0.003 | 5.80E-48 | 154.5 |  |  |  |  |  |
| 30 | rs12038026 | t | c | 0.765 | -0.01 | 0.002 | 2.26E-09 | 16.2 |  |  |  |  |  |
| 31 | rs17400787 | a | g | 0.1777 | -0.02 | 0.003 | 5.49E-11 | 52.6 |  |  |  |  |  |
| 32 | rs11590254 | a | t | 0.6886 | -0.02 | 0.002 | 4.34E-20 | 77.2 |  |  |  |  |  |
| 33 | rs12733767 | t | c | 0.1024 | -0.02 | 0.003 | 9.38E-14 | 33.1 |  |  |  |  |  |
| 34 | rs12740374 | t | g | 0.2214 | 0.02 | 0.002 | 1.83E-10 | 62.1 |  |  |  |  |  |
| 35 | rs3768495 | t | c | 0.7173 | -0.02 | 0.002 | 1.07E-17 | 73.1 |  |  |  |  |  |
| 36 | rs12724682 | c | g | 0.1289 | 0.02 | 0.003 | 4.04E-13 | 40.4 |  |  |  |  |  |
| 37 | rs60804050 | a | g | 0.2559 | -0.02 | 0.002 | 5.01E-24 | 68.6 |  |  |  |  |  |
| 38 | rs57445715 | t | c | 0.7328 | 0.01 | 0.002 | 3.48E-12 | 17.6 |  |  |  |  |  |
| 39 | rs320826 | c | g | 0.4954 | 0.02 | 0.002 | 5.67E-12 | 90.1 | Excluded | Excluded | Excluded | Excluded | Excluded |
| 40 | rs9659073 | a | g | 0.4754 | -0.02 | 0.002 | 5.05E-26 | 89.8 |  |  |  |  |  |
| 41 | rs115161931 | t | c | 0.0402 | -0.04 | 0.005 | 4.62E-15 | 55.6 |  |  |  |  |  |
| 42 | rs905938 | t | c | 0.7351 | -0.04 | 0.002 | 8.43E-77 | 280.7 |  |  |  |  |  |
| 43 | rs61812150 | a | g | 0.0278 | 0.05 | 0.006 | 9.30E-18 | 60.9 |  |  |  |  |  |
| 44 | rs11264447 | t | g | 0.6552 | -0.01 | 0.002 | 1.29E-12 | 20.3 |  |  |  |  |  |
| 45 | rs822430 | a | g | 0.5221 | -0.01 | 0.002 | 4.15E-12 | 22.5 |  |  |  |  |  |
| 46 | rs6687139 | a | g | 0.9001 | -0.02 | 0.003 | 2.11E-13 | 32.4 |  |  |  |  |  |
| 47 | rs1052256 | a | g | 0.8118 | -0.02 | 0.002 | 3.15E-20 | 55.0 |  |  |  |  |  |
| 48 | rs78275727 | a | g | 0.7716 | 0.02 | 0.002 | 2.96E-13 | 63.5 |  |  |  |  |  |
| 49 | rs4233168 | a | t | 0.0812 | 0.02 | 0.003 | 1.09E-11 | 26.9 |  |  |  |  |  |
| 50 | rs11583447 | t | g | 0.5729 | -0.02 | 0.002 | 4.39E-26 | 88.2 |  |  |  |  |  |
| 51 | rs10913200 | a | g | 0.0285 | -0.05 | 0.006 | 2.06E-18 | 62.3 |  |  |  |  |  |
| 52 | rs576101 | a | t | 0.3249 | 0.02 | 0.002 | 9.66E-17 | 79.0 |  |  |  |  |  |
| 53 | rs3795503 | t | c | 0.3145 | 0.02 | 0.002 | 2.49E-18 | 77.7 |  |  |  |  |  |
| 54 | rs12071514 | a | c | 0.4106 | 0.02 | 0.002 | 1.03E-24 | 87.2 | rs10911240 | rs10911240 | rs10911240 | rs10911240 | rs10911240 |
| 55 | rs234640 | t | c | 0.5142 | -0.01 | 0.002 | 3.87E-12 | 22.5 |  |  |  |  |  |
| 56 | rs3850625 | a | g | 0.1182 | -0.03 | 0.003 | 3.42E-18 | 84.5 |  |  |  |  |  |
| 57 | rs72744832 | t | c | 0.9165 | 0.03 | 0.003 | 1.92E-18 | 62.0 |  |  |  |  |  |
| 58 | rs955865 | a | g | 0.4202 | 0.01 | 0.002 | 4.09E-15 | 21.9 |  |  |  |  |  |
| 59 | rs7536483 | a | t | 0.5726 | 0.02 | 0.002 | 7.49E-33 | 88.2 |  |  |  |  |  |
| 60 | rs7532674 | t | g | 0.261 | 0.02 | 0.002 | 2.70E-12 | 69.5 |  |  |  |  |  |
| 61 | rs6540718 | a | c | 0.0142 | 0.05 | 0.008 | 1.16E-12 | 31.5 |  |  |  |  |  |
| 62 | rs1415181 | t | c | 0.5926 | 0.01 | 0.002 | 6.85E-14 | 21.7 |  |  |  |  |  |
| 63 | rs11811226 | a | t | 0.5559 | -0.02 | 0.002 | 1.90E-16 | 88.9 |  |  |  |  |  |
| 64 | rs3121580 | t | c | 0.1728 | -0.02 | 0.002 | 3.28E-11 | 51.5 |  |  |  |  |  |
| 65 | rs6658835 | a | g | 0.7308 | -0.02 | 0.002 | 9.13E-20 | 70.9 |  |  |  |  |  |
| 66 | rs6604614 | c | g | 0.7154 | -0.02 | 0.002 | 5.13E-29 | 73.3 |  |  |  |  |  |
| 67 | rs78763889 | t | g | 0.0417 | 0.03 | 0.005 | 1.12E-12 | 32.4 |  |  |  |  |  |
| 68 | rs12724708 | a | t | 0.6426 | -0.02 | 0.002 | 1.66E-35 | 82.7 |  |  |  |  |  |
| 69 | rs61826465 | t | c | 0.7472 | 0.01 | 0.002 | 6.50E-11 | 17.0 |  |  |  |  |  |
| 70 | rs34780873 | t | g | 0.7971 | 0.02 | 0.002 | 2.58E-14 | 58.3 |  |  |  |  |  |
| 71 | rs2994330 | t | g | 0.197 | 0.02 | 0.002 | 6.62E-12 | 57.0 |  |  |  |  |  |
| 72 | rs114018835 | t | c | 0.0359 | 0.03 | 0.005 | 2.68E-09 | 28.1 |  |  |  |  |  |
| 73 | rs62106258 | t | c | 0.9514 | 0.05 | 0.004 | 4.20E-33 | 104.1 |  |  |  |  |  |
| 74 | rs7563362 | a | g | 0.1434 | -0.04 | 0.003 | 3.27E-39 | 177.0 |  |  |  |  |  |
| 75 | rs13402560 | c | g | 0.1869 | -0.02 | 0.002 | 1.43E-13 | 54.7 |  |  |  |  |  |
| 76 | rs12474969 | a | g | 0.3006 | 0.02 | 0.002 | 4.03E-16 | 75.7 |  |  |  |  |  |
| 77 | rs4668626 | a | g | 0.5839 | -0.01 | 0.002 | 2.35E-10 | 21.9 |  |  |  |  |  |
| 78 | rs6735681 | t | c | 0.8908 | 0.03 | 0.003 | 1.68E-19 | 78.8 |  |  |  |  |  |
| 79 | rs12713004 | a | g | 0.2746 | -0.04 | 0.002 | 2.40E-68 | 287.2 |  |  |  |  |  |
| 80 | rs1056074 | t | c | 0.8225 | 0.02 | 0.002 | 1.24E-11 | 52.6 |  |  |  |  |  |
| 81 | rs2118826 | a | t | 0.4899 | -0.02 | 0.002 | 1.48E-39 | 90.0 |  |  |  |  |  |
| 82 | rs11689727 | a | c | 0.3317 | -0.01 | 0.002 | 1.64E-09 | 20.0 |  |  |  |  |  |
| 83 | rs72799646 | a | g | 0.7718 | -0.02 | 0.002 | 1.19E-22 | 63.4 |  |  |  |  |  |
| 84 | rs1260326 | t | c | 0.3955 | -0.03 | 0.002 | 6.16E-64 | 193.8 |  |  |  |  |  |
| 85 | rs17496249 | a | g | 0.5546 | -0.01 | 0.002 | 6.92E-11 | 22.2 |  |  |  |  |  |
| 86 | rs5830612 | t | g | 0.8749 | -0.02 | 0.003 | 4.19E-11 | 39.4 | Excluded | Excluded | Excluded | Excluded | Excluded |
| 87 | rs10202845 | a | g | 0.8867 | 0.03 | 0.003 | 5.35E-22 | 81.4 |  |  |  |  |  |
| 88 | rs7593987 | a | g | 0.1914 | 0.02 | 0.002 | 1.03E-19 | 55.8 |  |  |  |  |  |
| 89 | rs11893991 | a | g | 0.4266 | 0.01 | 0.002 | 2.28E-09 | 22.0 |  |  |  |  |  |
| 90 | rs687914 | t | g | 0.2514 | -0.01 | 0.002 | 7.99E-11 | 16.9 |  |  |  |  |  |
| 91 | rs62136933 | a | g | 0.1842 | 0.03 | 0.002 | 9.01E-32 | 121.8 |  |  |  |  |  |
| 92 | rs2436772 | a | g | 0.2092 | 0.02 | 0.002 | 1.18E-15 | 59.6 |  |  |  |  |  |
| 93 | rs1875407 | t | c | 0.3812 | -0.01 | 0.002 | 8.41E-13 | 21.2 |  |  |  |  |  |
| 94 | rs79073127 | c | g | 0.1227 | -0.02 | 0.003 | 2.56E-15 | 38.8 |  |  |  |  |  |
| 95 | rs59985551 | t | c | 0.2261 | -0.03 | 0.002 | 2.43E-44 | 141.9 |  |  |  |  |  |
| 96 | rs1432559 | t | g | 0.7814 | -0.01 | 0.002 | 1.56E-09 | 15.4 |  |  |  |  |  |
| 97 | rs75022676 | a | g | 0.2079 | -0.02 | 0.002 | 2.84E-12 | 59.3 |  |  |  |  |  |
| 98 | rs7569084 | t | c | 0.5853 | -0.02 | 0.002 | 8.85E-16 | 87.4 |  |  |  |  |  |
| 99 | rs76517946 | a | c | 0.0814 | -0.04 | 0.004 | 1.69E-26 | 107.8 |  |  |  |  |  |
| 100 | rs6711815 | c | g | 0.1474 | 0.02 | 0.003 | 3.00E-09 | 45.3 |  |  |  |  |  |
| 101 | rs11545482 | t | c | 0.0205 | -0.04 | 0.007 | 3.57E-11 | 28.9 |  |  |  |  |  |
| 102 | rs4852257 | t | g | 0.4237 | 0.02 | 0.002 | 6.21E-34 | 88.0 |  |  |  |  |  |
| 103 | rs6749644 | t | g | 0.6871 | -0.01 | 0.002 | 6.46E-13 | 19.4 |  |  |  |  |  |
| 104 | rs13423761 | a | c | 0.327 | 0.01 | 0.002 | 5.62E-10 | 19.8 |  |  |  |  |  |
| 105 | rs6746854 | t | c | 0.8497 | -0.02 | 0.003 | 7.21E-10 | 46.0 |  |  |  |  |  |
| 106 | rs14976 | t | c | 0.3057 | 0.01 | 0.002 | 1.43E-12 | 19.1 |  |  |  |  |  |
| 107 | rs867529 | c | g | 0.2796 | 0.02 | 0.002 | 1.00E-18 | 72.6 |  |  |  |  |  |
| 108 | rs6738207 | a | g | 0.4007 | 0.01 | 0.002 | 4.15E-11 | 21.6 |  |  |  |  |  |
| 109 | rs10199020 | a | t | 0.494 | 0.01 | 0.002 | 2.87E-14 | 22.5 |  |  |  |  |  |
| 110 | rs201570119 | t | c | 0.7902 | -0.02 | 0.002 | 5.48E-17 | 59.7 | rs58584712 | rs58584712 | rs58584712 | rs58584712 | rs58584712 |
| 111 | rs55812028 | t | c | 0.2451 | 0.02 | 0.002 | 3.72E-13 | 66.7 |  |  |  |  |  |
| 112 | rs141033892 | a | g | 0.0504 | 0.03 | 0.004 | 2.16E-09 | 38.8 |  |  |  |  |  |
| 113 | rs12463948 | a | t | 0.8117 | 0.02 | 0.002 | 3.17E-13 | 55.1 |  |  |  |  |  |
| 114 | rs13392139 | a | g | 0.9347 | 0.03 | 0.004 | 1.87E-13 | 49.5 |  |  |  |  |  |
| 115 | rs4663096 | a | t | 0.1794 | 0.01 | 0.002 | 1.53E-09 | 13.3 |  |  |  |  | Outlier |
| 116 | rs3101041 | a | c | 0.7304 | -0.01 | 0.002 | 2.97E-10 | 17.7 | Excluded | Excluded | Excluded | Excluded | Excluded |
| 117 | rs1128249 | t | g | 0.3919 | -0.02 | 0.002 | 2.89E-25 | 85.9 |  |  |  |  |  |
| 118 | rs2102808 | t | g | 0.1193 | 0.02 | 0.003 | 1.22E-11 | 37.8 |  |  |  |  |  |
| 119 | rs488621 | a | g | 0.5312 | -0.02 | 0.002 | 2.86E-24 | 89.7 |  |  |  |  |  |
| 120 | rs12151587 | t | c | 0.5811 | 0.02 | 0.002 | 6.21E-24 | 87.7 |  |  |  |  |  |
| 121 | rs2111459 | t | c | 0.2425 | 0.01 | 0.002 | 1.95E-11 | 16.5 |  |  |  |  |  |
| 122 | rs55852614 | t | c | 0.7526 | 0.04 | 0.002 | 3.29E-73 | 268.4 |  |  |  |  |  |
| 123 | rs16862227 | t | g | 0.2301 | -0.02 | 0.002 | 4.80E-19 | 63.8 |  |  |  |  |  |
| 124 | rs741610 | a | g | 0.6691 | -0.01 | 0.002 | 8.09E-14 | 19.9 |  |  |  |  |  |
| 125 | rs2444563 | t | c | 0.7921 | 0.02 | 0.002 | 8.86E-16 | 59.3 |  |  |  |  |  |
| 126 | rs112405294 | a | g | 0.0821 | 0.03 | 0.003 | 8.49E-14 | 61.1 |  |  |  |  |  |
| 127 | rs137968292 | t | c | 0.011 | 0.06 | 0.009 | 3.16E-10 | 35.3 |  |  |  |  |  |
| 128 | rs295139 | a | t | 0.4129 | 0.01 | 0.002 | 1.31E-13 | 21.8 |  |  |  | Outlier |  |
| 129 | rs72931013 | a | c | 0.9792 | -0.04 | 0.007 | 5.52E-10 | 29.3 |  |  |  |  |  |
| 130 | rs72938315 | c | g | 0.8549 | -0.02 | 0.003 | 6.68E-15 | 44.7 |  |  | Outlier |  |  |
| 131 | rs62195405 | a | g | 0.4259 | 0.01 | 0.002 | 9.14E-15 | 22.0 |  |  |  |  |  |
| 132 | rs1320929 | a | g | 0.4367 | 0.01 | 0.002 | 6.09E-13 | 22.2 |  |  |  |  |  |
| 133 | rs1047891 | a | c | 0.3159 | 0.02 | 0.002 | 5.70E-31 | 77.9 |  |  |  |  |  |
| 134 | rs6754292 | t | c | 0.6446 | 0.02 | 0.002 | 1.65E-21 | 82.5 |  |  |  |  |  |
| 135 | rs12622189 | a | g | 0.7422 | 0.03 | 0.002 | 1.34E-35 | 155.1 |  |  |  |  |  |
| 136 | rs76018285 | a | g | 0.9417 | -0.03 | 0.004 | 1.84E-13 | 44.5 |  |  |  |  |  |
| 137 | rs10194082 | t | c | 0.5897 | 0.02 | 0.002 | 2.64E-27 | 87.2 |  |  |  |  |  |
| 138 | rs145296160 | t | c | 0.9829 | 0.06 | 0.008 | 4.44E-15 | 54.5 |  |  |  |  |  |
| 139 | rs6711710 | t | c | 0.2188 | -0.03 | 0.002 | 8.28E-40 | 138.6 |  |  |  |  |  |
| 140 | rs2629047 | a | t | 0.3943 | -0.02 | 0.002 | 2.08E-18 | 86.0 |  |  |  |  |  |
| 141 | rs139441245 | a | t | 0.0109 | -0.06 | 0.009 | 1.71E-10 | 35.0 | Excluded |  | Excluded | Excluded |  |
| 142 | rs1522802 | a | c | 0.2181 | 0.02 | 0.002 | 5.41E-13 | 61.4 |  |  |  |  |  |
| 143 | rs17246129 | a | g | 0.3044 | 0.03 | 0.002 | 1.27E-35 | 171.7 |  |  |  |  |  |
| 144 | rs41265094 | c | g | 0.9853 | -0.06 | 0.007 | 1.06E-17 | 47.0 | Excluded |  | Excluded | Excluded | Excluded |
| 145 | rs4973392 | a | g | 0.2268 | -0.02 | 0.002 | 1.63E-20 | 63.2 |  |  |  |  |  |
| 146 | rs10202701 | t | c | 0.5419 | 0.02 | 0.002 | 3.11E-33 | 89.4 |  |  |  |  |  |
| 147 | rs73000823 | t | c | 0.1563 | -0.02 | 0.003 | 1.74E-15 | 47.5 |  |  |  |  |  |
| 148 | rs3116201 | a | g | 0.0977 | -0.03 | 0.003 | 2.98E-21 | 71.5 |  |  |  |  |  |
| 149 | rs79057767 | a | g | 0.9677 | -0.05 | 0.005 | 3.32E-21 | 70.4 |  |  |  |  |  |
| 150 | rs6755070 | t | g | 0.3501 | -0.02 | 0.002 | 2.04E-23 | 82.0 | rs6724117 | rs6724117 | rs6724117 | rs6724117 | rs6724117 |
| 151 | rs2971857 | a | g | 0.5752 | -0.01 | 0.002 | 3.70E-10 | 22.0 |  |  |  |  |  |
| 152 | rs12476059 | t | c | 0.8495 | 0.02 | 0.003 | 4.65E-10 | 46.1 |  |  |  |  |  |
| 153 | rs61277584 | a | g | 0.4733 | 0.01 | 0.002 | 2.64E-13 | 22.4 |  |  |  |  |  |
| 154 | rs2310876 | a | g | 0.6693 | 0.01 | 0.002 | 2.89E-12 | 19.9 |  |  |  |  |  |
| 155 | rs6718026 | t | c | 0.5857 | -0.02 | 0.002 | 2.46E-19 | 87.4 |  |  |  |  |  |
| 156 | rs6763944 | t | c | 0.7234 | 0.01 | 0.002 | 5.20E-11 | 18.0 |  |  |  |  |  |
| 157 | rs2270894 | c | g | 0.7966 | 0.03 | 0.002 | 1.25E-42 | 131.4 |  |  |  |  |  |
| 158 | rs12633841 | t | g | 0.1778 | 0.02 | 0.003 | 6.40E-13 | 52.7 |  |  |  |  |  |
| 159 | rs7610055 | a | g | 0.1207 | -0.04 | 0.003 | 3.55E-38 | 153.0 |  |  |  |  |  |
| 160 | rs7615332 | a | g | 0.4221 | 0.02 | 0.002 | 2.48E-26 | 87.9 |  |  |  |  |  |
| 161 | rs73040019 | t | c | 0.8419 | -0.02 | 0.003 | 1.54E-14 | 47.9 |  |  |  |  |  |
| 162 | rs6550846 | t | c | 0.7895 | 0.02 | 0.002 | 2.07E-15 | 59.9 |  |  |  |  |  |
| 163 | rs4858605 | t | c | 0.1848 | 0.02 | 0.002 | 5.02E-11 | 54.3 | Outlier |  |  |  |  |
| 164 | rs4858697 | a | g | 0.4184 | -0.01 | 0.002 | 3.06E-14 | 21.9 |  |  |  |  |  |
| 165 | rs591668 | a | g | 0.396 | -0.02 | 0.002 | 2.00E-19 | 86.2 |  |  |  |  |  |
| 166 | rs77156651 | a | g | 0.0537 | -0.03 | 0.004 | 6.83E-16 | 41.2 |  |  |  |  |  |
| 167 | rs72858391 | a | g | 0.0271 | 0.04 | 0.006 | 1.33E-14 | 38.0 |  |  |  |  |  |
| 168 | rs9838614 | t | g | 0.6119 | 0.02 | 0.002 | 1.21E-21 | 85.6 |  |  |  |  |  |
| 169 | rs13067055 | a | g | 0.3506 | 0.01 | 0.002 | 1.51E-11 | 20.5 |  |  |  |  |  |
| 170 | rs34345884 | c | g | 0.0214 | -0.04 | 0.007 | 6.13E-10 | 30.2 |  |  |  |  |  |
| 171 | rs17606778 | a | c | 0.1959 | 0.02 | 0.002 | 2.86E-12 | 56.7 |  |  |  |  |  |
| 172 | rs140440099 | a | g | 0.0234 | 0.06 | 0.006 | 1.45E-22 | 74.1 |  |  |  |  |  |
| 173 | rs11717749 | t | c | 0.1147 | 0.03 | 0.003 | 5.30E-24 | 82.3 |  |  |  |  |  |
| 174 | rs114718455 | a | g | 0.9651 | -0.04 | 0.005 | 3.54E-12 | 48.5 |  |  |  |  |  |
| 175 | rs11130474 | c | g | 0.4618 | 0.02 | 0.002 | 1.56E-19 | 89.5 |  |  |  |  |  |
| 176 | rs6762851 | t | c | 0.6427 | 0.02 | 0.002 | 1.50E-28 | 82.7 |  |  |  |  |  |
| 177 | rs550944 | a | t | 0.6772 | -0.01 | 0.002 | 3.09E-09 | 19.7 |  |  |  |  |  |
| 178 | rs9809116 | a | g | 0.5919 | 0.02 | 0.002 | 1.31E-16 | 87.0 |  |  |  |  |  |
| 179 | rs7633464 | a | g | 0.4786 | 0.02 | 0.002 | 1.28E-20 | 89.9 |  |  |  | Outlier |  |
| 180 | rs13088318 | a | g | 0.6631 | -0.02 | 0.002 | 1.15E-17 | 80.5 |  |  |  |  |  |
| 181 | rs4073154 | a | g | 0.2216 | -0.03 | 0.002 | 1.93E-33 | 139.8 |  |  |  |  |  |
| 182 | rs546819639 | t | c | 0.0936 | 0.03 | 0.003 | 1.05E-18 | 68.8 | rs7622053 | rs7622053 | rs7622053 | rs7622053 | rs7622053 |
| 183 | rs9832919 | a | g | 0.6426 | 0.02 | 0.002 | 7.90E-20 | 82.7 |  |  |  |  |  |
| 184 | rs9883955 | t | g | 0.373 | 0.02 | 0.002 | 4.63E-21 | 84.3 |  |  |  |  |  |
| 185 | rs2871960 | a | c | 0.5552 | -0.05 | 0.002 | 2.17E-135 | 556.6 |  |  |  |  |  |
| 186 | rs35408555 | a | g | 0.7718 | 0.01 | 0.002 | 1.76E-09 | 15.9 |  |  |  |  |  |
| 187 | rs74497536 | a | g | 0.0232 | 0.04 | 0.006 | 1.56E-09 | 32.7 |  |  |  |  |  |
| 188 | rs900399 | a | g | 0.6014 | -0.02 | 0.002 | 1.35E-17 | 86.4 |  |  |  |  |  |
| 189 | rs1048243 | a | g | 0.635 | -0.01 | 0.002 | 2.62E-14 | 20.9 |  |  |  |  |  |
| 190 | rs2115959 | a | c | 0.4543 | -0.01 | 0.002 | 5.70E-14 | 22.3 |  |  |  |  |  |
| 191 | rs9647379 | c | g | 0.4111 | 0.02 | 0.002 | 2.75E-21 | 87.2 |  |  |  |  |  |
| 192 | rs4894532 | t | g | 0.3648 | 0.03 | 0.002 | 1.53E-45 | 187.9 |  |  |  |  |  |
| 193 | rs843342 | t | c | 0.4809 | -0.01 | 0.002 | 1.06E-09 | 22.5 |  |  |  |  |  |
| 194 | rs11545169 | t | g | 0.1613 | -0.04 | 0.003 | 8.19E-43 | 195.0 |  |  |  |  |  |
| 195 | rs2194411 | a | g | 0.1283 | 0.04 | 0.003 | 2.43E-54 | 161.2 |  |  |  |  |  |
| 196 | rs61732778 | a | g | 0.071 | 0.02 | 0.004 | 3.13E-10 | 23.8 |  |  |  |  |  |
| 197 | rs62288215 | a | c | 0.6078 | 0.01 | 0.002 | 1.66E-09 | 21.5 |  |  |  |  |  |
| 198 | rs6837180 | a | g | 0.4163 | -0.01 | 0.002 | 2.56E-10 | 21.9 |  |  |  |  |  |
| 199 | rs111391498 | a | g | 0.9523 | 0.03 | 0.004 | 3.65E-14 | 36.8 |  |  |  |  |  |
| 200 | rs7680647 | t | c | 0.6438 | -0.02 | 0.002 | 2.26E-20 | 82.6 |  |  |  |  |  |
| 201 | rs59950280 | a | g | 0.3316 | -0.03 | 0.002 | 7.32E-36 | 179.7 |  |  |  |  |  |
| 202 | rs183275998 | c | g | 0.2842 | 0.01 | 0.002 | 8.20E-11 | 18.3 |  |  |  |  |  |
| 203 | rs62288581 | a | g | 0.4206 | -0.01 | 0.002 | 2.54E-12 | 21.9 |  |  |  |  |  |
| 204 | rs223929 | a | c | 0.7179 | 0.02 | 0.002 | 1.67E-20 | 73.0 |  |  |  |  |  |
| 205 | rs1472852 | a | c | 0.1582 | -0.06 | 0.003 | 8.22E-135 | 432.1 |  |  |  |  |  |
| 206 | rs60799144 | a | t | 0.4001 | 0.01 | 0.002 | 1.16E-11 | 21.6 | Excluded | Excluded | Excluded | Excluded | Excluded |
| 207 | rs2324154 | a | c | 0.5096 | 0.02 | 0.002 | 1.92E-15 | 90.0 |  |  |  |  |  |
| 208 | rs74416750 | t | c | 0.1497 | -0.02 | 0.003 | 4.65E-15 | 45.9 |  |  |  |  |  |
| 209 | rs781669 | t | c | 0.5236 | 0.02 | 0.002 | 3.14E-18 | 89.9 |  |  |  |  |  |
| 210 | rs139921635 | t | g | 0.0236 | 0.04 | 0.006 | 2.66E-10 | 33.2 |  |  |  |  |  |
| 211 | rs58083390 | t | c | 0.0708 | -0.03 | 0.004 | 3.35E-18 | 53.3 | rs79863790 | rs79863790 | rs79863790 | rs79863790 | rs79863790 |
| 212 | rs112948778 | t | c | 0.0169 | 0.07 | 0.007 | 1.36E-22 | 73.3 |  |  |  |  |  |
| 213 | rs1443536 | a | g | 0.695 | -0.02 | 0.002 | 1.91E-26 | 76.4 |  |  |  |  |  |
| 214 | rs17010957 | t | c | 0.8515 | 0.02 | 0.003 | 6.34E-20 | 45.6 |  |  |  |  |  |
| 215 | rs1841738 | a | g | 0.4817 | 0.02 | 0.002 | 1.32E-24 | 89.9 |  |  |  |  |  |
| 216 | rs62306393 | a | c | 0.6467 | -0.01 | 0.002 | 1.17E-11 | 20.6 |  |  |  |  |  |
| 217 | rs78896073 | a | t | 0.0464 | -0.03 | 0.005 | 2.90E-10 | 35.9 |  |  |  |  |  |
| 218 | rs143847362 | a | g | 0.9745 | 0.04 | 0.006 | 2.65E-12 | 35.8 |  |  |  |  |  |
| 219 | rs1493132 | t | c | 0.6614 | -0.01 | 0.002 | 3.29E-11 | 20.2 |  |  |  |  |  |
| 220 | rs10007409 | t | c | 0.3101 | -0.02 | 0.002 | 7.11E-22 | 77.1 |  |  |  |  |  |
| 221 | rs6821305 | a | c | 0.601 | -0.02 | 0.002 | 3.15E-26 | 86.4 |  |  |  |  |  |
| 222 | rs10518426 | t | c | 0.5838 | -0.02 | 0.002 | 3.49E-22 | 87.5 |  |  |  |  |  |
| 223 | rs7689420 | t | c | 0.1688 | -0.05 | 0.003 | 1.50E-76 | 316.1 |  |  |  |  |  |
| 224 | rs116662954 | a | g | 0.0274 | -0.05 | 0.006 | 8.75E-19 | 60.0 |  |  |  |  |  |
| 225 | rs7679276 | a | g | 0.0456 | 0.03 | 0.005 | 5.93E-12 | 35.3 |  |  |  |  |  |
| 226 | rs7692387 | a | g | 0.1854 | 0.02 | 0.002 | 3.53E-12 | 54.4 |  |  |  |  |  |
| 227 | rs73856768 | t | c | 0.9195 | 0.02 | 0.004 | 1.55E-12 | 26.7 |  |  |  |  |  |
| 228 | rs395980 | t | g | 0.7365 | 0.02 | 0.002 | 1.02E-17 | 69.9 |  |  |  |  |  |
| 229 | rs6552198 | a | g | 0.6378 | -0.02 | 0.002 | 7.18E-18 | 83.2 |  |  |  |  |  |
| 230 | rs10015974 | a | g | 0.2407 | -0.02 | 0.002 | 1.10E-15 | 65.8 |  |  |  |  |  |
| 231 | rs2578557 | t | c | 0.6417 | -0.01 | 0.002 | 1.99E-12 | 20.7 |  |  |  |  |  |
| 232 | rs9716700 | a | c | 0.1901 | -0.02 | 0.003 | 1.36E-17 | 55.5 |  |  |  |  |  |
| 233 | rs12656497 | t | c | 0.4037 | 0.03 | 0.002 | 1.22E-40 | 195.2 |  |  |  |  |  |
| 234 | rs7718953 | a | c | 0.7352 | 0.02 | 0.002 | 4.44E-15 | 70.1 |  |  |  |  |  |
| 235 | rs6897259 | t | c | 0.3779 | -0.01 | 0.002 | 9.58E-14 | 21.2 |  |  |  |  |  |
| 236 | rs7731023 | a | g | 0.4251 | -0.02 | 0.002 | 3.48E-18 | 88.0 |  |  |  |  |  |
| 237 | rs1818782 | a | c | 0.3437 | -0.02 | 0.002 | 3.94E-14 | 81.3 |  |  |  |  |  |
| 238 | rs73087475 | t | c | 0.9077 | -0.03 | 0.003 | 3.07E-18 | 67.9 |  |  |  |  |  |
| 239 | rs62372052 | a | g | 0.8897 | -0.04 | 0.003 | 8.25E-40 | 141.4 |  |  |  |  |  |
| 240 | rs62370472 | t | c | 0.791 | 0.03 | 0.002 | 1.45E-27 | 134.0 |  |  |  |  |  |
| 241 | rs1833888 | t | c | 0.2322 | -0.02 | 0.002 | 7.93E-21 | 64.2 |  |  |  |  |  |
| 242 | rs35748083 | t | c | 0.5682 | -0.02 | 0.002 | 9.77E-25 | 88.4 |  |  |  |  |  |
| 243 | rs4865956 | a | t | 0.6965 | -0.03 | 0.002 | 3.85E-36 | 171.4 |  |  |  |  |  |
| 244 | rs465983 | a | g | 0.7642 | 0.02 | 0.002 | 4.75E-12 | 64.9 |  |  |  |  |  |
| 245 | rs282302 | a | g | 0.0902 | 0.03 | 0.003 | 6.51E-14 | 66.5 |  |  |  |  |  |
| 246 | rs34287 | a | g | 0.341 | 0.02 | 0.002 | 1.17E-20 | 81.0 |  |  |  |  |  |
| 247 | rs152353 | a | g | 0.199 | 0.02 | 0.002 | 4.05E-12 | 57.4 |  |  |  |  |  |
| 248 | rs10036789 | c | g | 0.5391 | -0.02 | 0.002 | 1.17E-17 | 89.5 |  |  |  |  |  |
| 249 | rs609659 | a | g | 0.5761 | -0.01 | 0.002 | 7.55E-13 | 22.0 |  |  |  |  |  |
| 250 | rs6877994 | c | g | 0.4842 | -0.01 | 0.002 | 9.86E-11 | 22.5 |  |  |  |  |  |
| 251 | rs115912456 | a | g | 0.9588 | -0.06 | 0.005 | 3.69E-34 | 128.1 |  |  |  |  |  |
| 252 | rs13178887 | t | c | 0.6133 | 0.02 | 0.002 | 2.85E-17 | 85.4 |  |  |  |  |  |
| 253 | rs2367279 | t | g | 0.9047 | -0.02 | 0.003 | 3.82E-09 | 31.1 |  |  |  |  |  |
| 254 | rs55838622 | a | c | 0.754 | -0.02 | 0.002 | 1.24E-16 | 66.8 |  |  |  |  |  |
| 255 | rs261223 | a | c | 0.6298 | -0.02 | 0.002 | 2.30E-19 | 84.0 |  |  |  |  |  |
| 256 | rs331917 | a | g | 0.4197 | 0.01 | 0.002 | 3.54E-11 | 21.9 |  |  |  |  |  |
| 257 | rs861674 | a | t | 0.5422 | -0.01 | 0.002 | 1.37E-11 | 22.4 |  |  |  |  |  |
| 258 | rs9327336 | t | c | 0.6565 | -0.01 | 0.002 | 1.78E-10 | 20.3 |  |  |  |  |  |
| 259 | rs7729769 | a | g | 0.5048 | 0.01 | 0.002 | 4.56E-11 | 22.5 | Excluded | Excluded | Excluded | Excluded | Excluded |
| 260 | rs6860245 | c | g | 0.248 | 0.06 | 0.002 | 9.66E-160 | 605.4 |  |  |  |  |  |
| 261 | rs7735162 | t | c | 0.7244 | 0.02 | 0.002 | 2.57E-13 | 71.9 |  |  |  |  |  |
| 262 | rs6866701 | a | g | 0.076 | 0.03 | 0.004 | 8.01E-18 | 56.9 |  |  |  |  |  |
| 263 | rs1291602 | t | c | 0.1592 | -0.03 | 0.003 | 3.42E-32 | 108.5 |  |  |  |  |  |
| 264 | rs7735891 | t | c | 0.4629 | 0.03 | 0.002 | 1.14E-42 | 201.6 |  |  |  |  |  |
| 265 | rs4976261 | c | g | 0.2499 | -0.03 | 0.002 | 8.67E-43 | 152.0 |  |  |  |  |  |
| 266 | rs639933 | a | c | 0.6333 | 0.01 | 0.002 | 8.14E-13 | 20.9 |  |  |  |  |  |
| 267 | rs11242236 | a | g | 0.4533 | -0.01 | 0.002 | 1.84E-14 | 22.3 |  |  |  |  |  |
| 268 | rs12055045 | t | c | 0.2062 | 0.02 | 0.002 | 7.15E-20 | 59.0 |  |  |  |  |  |
| 269 | rs3822742 | a | c | 0.3706 | 0.02 | 0.002 | 1.03E-16 | 84.0 |  |  |  |  |  |
| 270 | rs153662 | t | g | 0.5886 | 0.01 | 0.002 | 1.23E-12 | 21.8 |  |  |  |  |  |
| 271 | rs853170 | t | c | 0.7398 | -0.02 | 0.002 | 6.04E-13 | 69.3 |  |  |  |  |  |
| 272 | rs10069931 | t | c | 0.3282 | -0.01 | 0.002 | 3.28E-13 | 19.9 |  |  |  |  |  |
| 273 | rs13170063 | a | g | 0.5921 | -0.02 | 0.002 | 4.11E-15 | 87.0 |  |  |  |  |  |
| 274 | rs4073717 | t | g | 0.2016 | -0.03 | 0.002 | 5.85E-34 | 130.5 |  |  |  |  |  |
| 275 | rs33852 | a | g | 0.6771 | -0.02 | 0.002 | 1.04E-24 | 78.8 |  |  |  |  |  |
| 276 | rs75767302 | a | g | 0.0441 | 0.03 | 0.005 | 8.52E-13 | 34.2 |  |  |  |  |  |
| 277 | rs6874142 | t | g | 0.8862 | -0.02 | 0.003 | 1.68E-14 | 36.3 |  |  |  |  |  |
| 278 | rs6881592 | a | g | 0.6347 | 0.02 | 0.002 | 2.81E-21 | 83.5 |  |  |  |  |  |
| 279 | rs7733195 | a | g | 0.3494 | -0.02 | 0.002 | 4.49E-21 | 81.9 |  |  |  |  |  |
| 280 | rs244711 | t | c | 0.686 | 0.03 | 0.002 | 1.54E-37 | 174.6 |  |  |  |  |  |
| 281 | rs13177762 | t | c | 0.1374 | 0.03 | 0.003 | 4.08E-20 | 96.1 |  |  |  |  |  |
| 282 | rs62398471 | a | g | 0.2946 | 0.02 | 0.002 | 1.16E-17 | 74.9 |  |  |  |  |  |
| 283 | rs4959697 | t | c | 0.7435 | -0.01 | 0.002 | 4.29E-10 | 17.2 | Excluded | Excluded | Excluded | Excluded | Excluded |
| 284 | rs9379084 | a | g | 0.1155 | -0.03 | 0.003 | 1.84E-20 | 82.8 |  |  |  |  |  |
| 285 | rs11243202 | t | c | 0.514 | -0.03 | 0.002 | 2.83E-57 | 202.5 |  |  |  |  |  |
| 286 | rs6923462 | t | c | 0.8429 | -0.03 | 0.003 | 3.41E-24 | 107.3 |  |  |  |  |  |
| 287 | rs35947355 | a | g | 0.224 | -0.02 | 0.002 | 5.59E-15 | 62.6 |  |  |  |  |  |
| 288 | rs2142644 | a | c | 0.672 | -0.02 | 0.002 | 3.37E-19 | 79.4 |  |  |  |  |  |
| 289 | rs41271299 | t | c | 0.0513 | 0.06 | 0.004 | 3.19E-47 | 157.8 |  |  |  |  |  |
| 290 | rs9379832 | a | g | 0.7447 | 0.02 | 0.002 | 3.77E-24 | 68.5 |  |  |  |  |  |
| 291 | rs17767294 | a | g | 0.9226 | 0.02 | 0.004 | 1.94E-10 | 25.7 |  |  |  |  |  |
| 292 | rs3095275 | a | g | 0.1998 | 0.02 | 0.002 | 8.96E-21 | 57.6 | Excluded | Excluded | Excluded | Excluded |  |
| 293 | rs9266244 | a | g | 0.7077 | -0.04 | 0.002 | 1.21E-94 | 298.2 |  |  |  |  |  |
| 294 | rs184318723 | c | g | 0.0466 | 0.06 | 0.007 | 3.18E-21 | 144.1 |  |  | Excluded |  |  |
| 295 | rs9276931 | a | g | 0.8852 | -0.03 | 0.003 | 4.65E-23 | 82.4 | Excluded | Excluded | Excluded | Excluded |  |
| 296 | rs1705001 | a | g | 0.2413 | 0.01 | 0.002 | 3.66E-11 | 16.5 | Excluded | Excluded | Excluded | Excluded |  |
| 297 | rs386906 | t | c | 0.6533 | 0.02 | 0.002 | 6.55E-27 | 81.6 |  |  |  |  |  |
| 298 | rs688209 | t | g | 0.8021 | 0.02 | 0.002 | 1.26E-16 | 57.2 |  |  |  |  |  |
| 299 | rs61431557 | t | c | 0.1368 | 0.04 | 0.003 | 3.83E-50 | 170.2 |  |  |  |  |  |
| 300 | rs3734254 | t | c | 0.7909 | 0.03 | 0.002 | 2.32E-33 | 134.1 |  |  |  |  |  |
| 301 | rs72894776 | t | c | 0.1245 | 0.02 | 0.003 | 1.74E-15 | 39.3 |  |  |  |  |  |
| 302 | rs9394578 | a | c | 0.2577 | -0.02 | 0.002 | 2.65E-14 | 68.9 |  |  |  |  |  |
| 303 | rs33966734 | a | c | 0.0136 | -0.11 | 0.008 | 1.89E-41 | 146.2 |  |  |  |  |  |
| 304 | rs7762731 | a | g | 0.8604 | 0.02 | 0.003 | 3.91E-11 | 43.3 |  |  |  |  |  |
| 305 | rs1324538 | a | t | 0.3836 | 0.02 | 0.002 | 1.73E-34 | 85.2 |  |  |  |  |  |
| 306 | rs2208605 | t | c | 0.2078 | 0.01 | 0.002 | 3.62E-10 | 14.8 |  |  |  |  |  |
| 307 | rs6931421 | t | g | 0.6776 | 0.03 | 0.002 | 2.31E-43 | 177.1 |  |  |  |  |  |
| 308 | rs9350850 | t | c | 0.9204 | -0.03 | 0.003 | 2.92E-24 | 59.4 |  |  |  |  |  |
| 309 | rs34951006 | t | g | 0.137 | 0.02 | 0.003 | 3.46E-13 | 42.6 |  |  |  |  |  |
| 310 | rs194633 | a | c | 0.4417 | -0.01 | 0.002 | 1.62E-14 | 22.2 |  |  |  |  |  |
| 311 | rs3813498 | t | c | 0.8126 | 0.03 | 0.002 | 6.35E-29 | 123.4 |  |  |  |  |  |
| 312 | rs1405212 | t | c | 0.3721 | -0.02 | 0.002 | 1.10E-21 | 84.2 |  |  |  |  |  |
| 313 | rs3966775 | a | c | 0.528 | 0.02 | 0.002 | 6.65E-26 | 89.8 |  |  |  |  |  |
| 314 | rs9388490 | t | c | 0.4394 | 0.05 | 0.002 | 1.33E-130 | 555.2 |  |  |  |  |  |
| 315 | rs13212016 | t | c | 0.2707 | -0.02 | 0.002 | 1.75E-21 | 71.1 |  |  |  |  |  |
| 316 | rs72959041 | a | g | 0.0494 | 0.04 | 0.004 | 1.56E-18 | 67.7 |  |  |  |  |  |
| 317 | rs9321170 | a | g | 0.7272 | 0.02 | 0.002 | 1.15E-16 | 71.5 |  |  |  |  |  |
| 318 | rs7740107 | a | t | 0.7362 | -0.05 | 0.002 | 7.46E-99 | 437.6 |  |  |  |  |  |
| 319 | rs78051210 | t | c | 0.9229 | -0.03 | 0.004 | 1.64E-13 | 57.7 |  |  |  |  |  |
| 320 | rs6924144 | t | g | 0.7735 | -0.02 | 0.002 | 1.40E-14 | 63.1 |  |  |  |  |  |
| 321 | rs228433 | a | g | 0.6181 | -0.01 | 0.002 | 4.90E-13 | 21.3 |  |  |  |  |  |
| 322 | rs2223966 | a | g | 0.7651 | 0.02 | 0.002 | 5.63E-17 | 64.7 |  |  |  |  |  |
| 323 | rs35686278 | a | g | 0.2265 | 0.01 | 0.002 | 8.96E-11 | 15.8 |  |  |  |  |  |
| 324 | rs9496306 | t | c | 0.3568 | -0.02 | 0.002 | 1.06E-14 | 82.7 |  |  |  |  |  |
| 325 | rs6570509 | t | g | 0.2868 | -0.02 | 0.002 | 1.27E-31 | 73.7 |  |  |  |  |  |
| 326 | rs12190712 | t | c | 0.0947 | 0.03 | 0.003 | 2.51E-17 | 69.5 |  |  |  |  |  |
| 327 | rs543650 | t | g | 0.4013 | -0.03 | 0.002 | 1.49E-37 | 194.8 |  |  |  |  |  |
| 328 | rs2635471 | a | g | 0.8541 | -0.02 | 0.003 | 5.04E-12 | 44.9 |  |  |  |  |  |
| 329 | rs3828729 | a | g | 0.6906 | 0.02 | 0.002 | 4.67E-15 | 77.0 |  |  |  |  |  |
| 330 | rs668871 | t | c | 0.4699 | 0.02 | 0.002 | 1.40E-27 | 89.7 |  |  |  |  |  |
| 331 | rs7768382 | t | c | 0.5229 | 0.02 | 0.002 | 1.57E-26 | 89.9 |  |  |  |  |  |
| 332 | rs2763263 | a | t | 0.2445 | -0.02 | 0.002 | 1.37E-14 | 66.5 |  |  |  |  |  |
| 333 | rs798548 | t | c | 0.6994 | 0.04 | 0.002 | 2.86E-68 | 303.1 |  |  |  |  |  |
| 334 | rs76400633 | t | c | 0.7831 | 0.02 | 0.002 | 5.92E-11 | 61.2 |  |  |  |  |  |
| 335 | rs12702693 | t | c | 0.4542 | 0.02 | 0.002 | 6.56E-20 | 89.3 |  |  |  |  |  |
| 336 | rs2389858 | a | t | 0.4945 | 0.02 | 0.002 | 1.68E-18 | 90.1 |  |  |  |  |  |
| 337 | rs12533452 | t | c | 0.1569 | 0.02 | 0.003 | 1.31E-19 | 47.7 |  |  |  |  |  |
| 338 | rs34776209 | t | c | 0.2475 | -0.03 | 0.002 | 1.78E-47 | 151.0 |  |  |  |  |  |
| 339 | rs2529049 | a | g | 0.2525 | 0.01 | 0.002 | 1.20E-10 | 17.0 |  |  |  |  |  |
| 340 | rs73085996 | c | g | 0.2319 | -0.01 | 0.002 | 5.43E-10 | 16.0 |  |  |  |  |  |
| 341 | rs12700667 | a | g | 0.7419 | -0.02 | 0.002 | 1.30E-17 | 69.0 |  |  |  |  |  |
| 342 | rs10951164 | c | g | 0.487 | 0.02 | 0.002 | 5.20E-16 | 90.0 |  |  |  |  |  |
| 343 | rs1708301 | c | g | 0.8509 | -0.02 | 0.003 | 1.99E-18 | 45.7 |  |  |  |  |  |
| 344 | rs177592 | c | g | 0.1013 | -0.02 | 0.003 | 6.83E-13 | 32.8 |  |  |  |  |  |
| 345 | rs62442203 | c | g | 0.4031 | -0.02 | 0.002 | 5.12E-23 | 86.7 |  |  |  |  |  |
| 346 | rs1717720 | a | g | 0.346 | -0.01 | 0.002 | 2.71E-12 | 20.4 |  |  |  |  |  |
| 347 | rs57734857 | a | t | 0.7958 | -0.02 | 0.002 | 5.85E-11 | 58.5 |  |  |  |  |  |
| 348 | rs10260816 | c | g | 0.5606 | -0.01 | 0.002 | 4.06E-13 | 22.2 |  |  |  |  |  |
| 349 | rs13237426 | t | c | 0.9381 | 0.04 | 0.004 | 1.24E-24 | 83.7 |  |  |  |  |  |
| 350 | rs723149 | a | g | 0.4372 | 0.03 | 0.002 | 1.43E-47 | 199.5 |  |  |  |  |  |
| 351 | rs2237474 | t | g | 0.3586 | 0.02 | 0.002 | 1.28E-20 | 82.9 |  |  |  |  |  |
| 352 | rs28895377 | a | t | 0.8847 | 0.02 | 0.003 | 2.36E-16 | 36.7 |  |  |  |  |  |
| 353 | rs12698934 | a | g | 0.1668 | 0.02 | 0.003 | 7.77E-15 | 50.1 |  |  |  |  |  |
| 354 | rs12540011 | a | g | 0.2835 | 0.02 | 0.002 | 3.01E-22 | 73.2 |  |  |  |  |  |
| 355 | rs12667702 | t | c | 0.2051 | -0.01 | 0.002 | 4.18E-10 | 14.7 | Excluded | Excluded | Excluded | Excluded | Excluded |
| 356 | rs10280623 | t | c | 0.803 | -0.02 | 0.002 | 2.73E-11 | 57.0 |  |  |  |  |  |
| 357 | rs78138888 | t | c | 0.0721 | -0.03 | 0.004 | 1.45E-13 | 54.2 |  |  |  |  |  |
| 358 | rs42039 | t | c | 0.2444 | 0.05 | 0.002 | 3.53E-106 | 416.1 | Outlier |  |  |  |  |
| 359 | rs3763467 | t | c | 0.8843 | 0.03 | 0.003 | 3.24E-18 | 82.9 |  |  |  |  |  |
| 360 | rs987666 | a | g | 0.1188 | 0.02 | 0.003 | 2.33E-10 | 37.7 |  |  |  |  |  |
| 361 | rs62621812 | a | g | 0.0204 | 0.07 | 0.007 | 3.16E-27 | 88.2 |  |  |  |  |  |
| 362 | rs9690338 | a | g | 0.9163 | -0.02 | 0.003 | 1.07E-11 | 27.6 |  |  |  |  |  |
| 363 | rs17480616 | c | g | 0.0307 | 0.04 | 0.005 | 4.50E-13 | 42.9 |  |  |  |  |  |
| 364 | rs199610373 | a | g | 0.8172 | -0.03 | 0.003 | 5.40E-27 | 121.1 | rs10435198 | rs10435198 | rs10435198 | rs10435198 | rs10435198 |
| 365 | rs269232 | a | g | 0.7042 | -0.01 | 0.002 | 1.17E-12 | 18.8 |  |  |  |  |  |
| 366 | rs822530 | a | t | 0.2047 | -0.03 | 0.002 | 2.36E-27 | 132.0 |  |  |  |  |  |
| 367 | rs6977416 | a | g | 0.3338 | 0.05 | 0.002 | 1.43E-113 | 501.2 |  |  |  |  |  |
| 368 | rs3918226 | t | c | 0.081 | -0.04 | 0.004 | 1.81E-27 | 107.3 |  |  |  |  |  |
| 369 | rs55911644 | a | t | 0.3609 | 0.01 | 0.002 | 8.93E-13 | 20.8 |  |  |  |  |  |
| 370 | rs75140902 | t | c | 0.0323 | -0.04 | 0.005 | 4.82E-15 | 45.0 |  |  |  |  |  |
| 371 | rs76364830 | a | g | 0.0634 | -0.05 | 0.004 | 2.70E-33 | 133.7 |  |  |  |  |  |
| 372 | rs7833274 | t | g | 0.3794 | 0.01 | 0.002 | 9.45E-10 | 21.2 |  |  |  |  |  |
| 373 | rs1063582 | t | g | 0.2351 | 0.02 | 0.002 | 1.12E-16 | 64.8 |  |  |  |  |  |
| 374 | rs62501195 | a | c | 0.8288 | 0.02 | 0.003 | 8.11E-15 | 51.1 |  |  |  |  |  |
| 375 | rs2321250 | t | c | 0.6429 | 0.01 | 0.002 | 2.91E-12 | 20.7 |  |  |  |  |  |
| 376 | rs6557894 | t | c | 0.4648 | -0.01 | 0.002 | 7.69E-11 | 22.4 |  |  |  |  |  |
| 377 | rs7816345 | t | c | 0.1682 | 0.03 | 0.003 | 6.23E-24 | 113.4 |  |  |  |  |  |
| 378 | rs6988165 | c | g | 0.573 | 0.01 | 0.002 | 4.18E-12 | 22.0 |  |  |  |  |  |
| 379 | rs6984699 | a | g | 0.7413 | -0.02 | 0.002 | 1.86E-24 | 69.1 |  |  |  |  |  |
| 380 | rs139424516 | c | g | 0.9783 | 0.04 | 0.007 | 1.49E-10 | 30.6 |  |  |  |  |  |
| 381 | rs72656010 | t | c | 0.8678 | 0.07 | 0.003 | 7.31E-126 | 506.8 |  |  |  |  |  |
| 382 | rs62515454 | t | c | 0.1822 | 0.04 | 0.002 | 1.59E-55 | 214.8 | Excluded | Excluded | Excluded | Excluded | Excluded |
| 383 | rs4737446 | t | g | 0.6944 | 0.02 | 0.002 | 4.90E-32 | 76.4 |  |  |  |  |  |
| 384 | rs16938625 | t | c | 0.8481 | -0.02 | 0.003 | 1.55E-13 | 46.4 |  |  |  |  |  |
| 385 | rs2925155 | t | c | 0.2612 | -0.02 | 0.002 | 5.47E-12 | 69.5 |  |  |  |  |  |
| 386 | rs61729527 | t | c | 0.0519 | -0.03 | 0.004 | 4.53E-16 | 39.9 |  |  |  |  |  |
| 387 | rs4735761 | a | c | 0.7143 | -0.03 | 0.002 | 3.66E-56 | 165.5 |  |  |  |  |  |
| 388 | rs7014590 | t | c | 0.7386 | 0.02 | 0.002 | 4.48E-26 | 69.6 |  |  |  |  |  |
| 389 | rs7829174 | a | g | 0.4637 | 0.01 | 0.002 | 2.24E-09 | 22.4 |  |  |  |  |  |
| 390 | rs2142331 | t | c | 0.6023 | -0.02 | 0.002 | 1.38E-17 | 86.3 |  |  |  |  |  |
| 391 | rs11778491 | c | g | 0.2518 | -0.02 | 0.002 | 4.05E-28 | 67.9 |  |  |  |  |  |
| 392 | rs10283100 | a | g | 0.0555 | -0.06 | 0.004 | 4.11E-44 | 170.0 |  |  |  |  |  |
| 393 | rs11779459 | t | c | 0.3545 | 0.01 | 0.002 | 1.37E-13 | 20.6 |  |  |  |  |  |
| 394 | rs4870941 | c | g | 0.238 | -0.03 | 0.002 | 1.09E-39 | 147.0 |  |  |  |  |  |
| 395 | rs1902789 | a | g | 0.7493 | -0.02 | 0.002 | 2.75E-27 | 67.7 |  |  |  |  |  |
| 396 | rs7816342 | a | g | 0.2326 | -0.03 | 0.002 | 4.08E-35 | 144.7 |  |  |  |  |  |
| 397 | rs4733571 | t | g | 0.6274 | -0.01 | 0.002 | 6.44E-13 | 21.1 |  |  |  |  |  |
| 398 | rs6987355 | a | t | 0.7991 | -0.02 | 0.002 | 1.80E-15 | 57.8 |  |  |  |  |  |
| 399 | rs12541381 | a | g | 0.2575 | -0.03 | 0.002 | 2.81E-49 | 155.0 |  |  |  |  |  |
| 400 | rs7821751 | a | c | 0.7369 | -0.02 | 0.002 | 2.54E-17 | 69.8 |  |  |  |  |  |
| 401 | rs12334478 | c | g | 0.5036 | 0.02 | 0.002 | 2.27E-17 | 90.1 |  |  |  |  |  |
| 402 | rs7015048 | t | c | 0.526 | 0.02 | 0.002 | 7.28E-16 | 89.8 |  |  |  |  |  |
| 403 | rs186474010 | a | g | 0.8088 | -0.02 | 0.003 | 1.09E-09 | 55.7 | Excluded | Excluded | Excluded | Excluded |  |
| 404 | rs73384223 | t | c | 0.8033 | 0.02 | 0.002 | 1.27E-17 | 56.9 |  |  |  |  |  |
| 405 | rs10815323 | a | g | 0.7963 | -0.02 | 0.002 | 2.72E-12 | 58.4 |  |  |  |  |  |
| 406 | rs56009005 | a | g | 0.0727 | -0.02 | 0.004 | 7.20E-11 | 24.3 |  |  |  |  |  |
| 407 | rs1341732 | a | g | 0.5814 | -0.01 | 0.002 | 7.23E-14 | 21.9 |  |  |  |  |  |
| 408 | rs6475052 | t | c | 0.411 | 0.01 | 0.002 | 5.60E-11 | 21.8 |  |  |  |  |  |
| 409 | rs7858712 | a | g | 0.0856 | -0.03 | 0.003 | 1.04E-24 | 63.4 |  |  |  |  |  |
| 410 | rs10963680 | a | g | 0.2436 | -0.01 | 0.002 | 2.96E-09 | 16.6 |  |  |  |  |  |
| 411 | rs12553324 | c | g | 0.5841 | -0.01 | 0.002 | 5.42E-13 | 21.9 |  |  |  |  |  |
| 412 | rs10814129 | a | g | 0.1654 | -0.02 | 0.003 | 1.01E-10 | 49.7 |  |  |  |  |  |
| 413 | rs13297831 | c | g | 0.5717 | 0.01 | 0.002 | 6.99E-13 | 22.1 |  |  |  |  |  |
| 414 | rs7029058 | t | c | 0.3625 | -0.01 | 0.002 | 1.71E-10 | 20.8 |  |  |  |  |  |
| 415 | rs1330826 | c | g | 0.2268 | 0.02 | 0.002 | 1.04E-12 | 63.2 |  |  |  |  |  |
| 416 | rs8181166 | c | g | 0.4997 | 0.01 | 0.002 | 8.57E-12 | 22.5 |  |  |  |  |  |
| 417 | rs12236754 | a | g | 0.1753 | -0.02 | 0.003 | 1.68E-22 | 52.1 |  |  |  |  |  |
| 418 | rs3901421 | c | g | 0.4871 | 0.02 | 0.002 | 7.55E-30 | 90.0 |  |  |  |  |  |
| 419 | rs10993370 | c | g | 0.5545 | -0.02 | 0.002 | 1.90E-19 | 89.0 |  |  |  | Outlier |  |
| 420 | rs2236406 | t | c | 0.6507 | -0.04 | 0.002 | 1.26E-87 | 327.7 |  |  |  |  |  |
| 421 | rs1984119 | t | c | 0.7441 | 0.02 | 0.002 | 1.27E-26 | 68.6 |  |  |  |  |  |
| 422 | rs6477489 | a | c | 0.7793 | -0.04 | 0.002 | 5.17E-59 | 247.9 |  |  |  |  |  |
| 423 | rs188738880 | t | c | 0.9605 | -0.04 | 0.005 | 6.97E-19 | 54.7 |  |  |  |  |  |
| 424 | rs10819542 | a | g | 0.6336 | 0.01 | 0.002 | 2.04E-12 | 20.9 |  |  |  |  |  |
| 425 | rs902144 | c | g | 0.4806 | 0.01 | 0.002 | 7.24E-13 | 22.5 |  |  |  |  |  |
| 426 | rs7849060 | t | c | 0.71 | -0.03 | 0.002 | 1.48E-35 | 166.9 |  |  |  |  |  |
| 427 | rs1341215 | a | g | 0.1374 | 0.02 | 0.003 | 6.32E-17 | 42.7 |  |  |  |  |  |
| 428 | rs1008158 | a | g | 0.6581 | -0.02 | 0.002 | 3.90E-15 | 81.1 |  |  |  |  |  |
| 429 | rs1249741 | a | t | 0.4759 | -0.01 | 0.002 | 1.77E-13 | 22.5 |  |  |  |  |  |
| 430 | rs5010187 | a | g | 0.5008 | 0.01 | 0.002 | 6.45E-12 | 22.5 |  |  |  |  |  |
| 431 | rs12344772 | t | g | 0.0832 | -0.04 | 0.004 | 2.00E-36 | 109.9 |  |  |  |  |  |
| 432 | rs115535131 | c | g | 0.0118 | -0.05 | 0.009 | 1.54E-09 | 26.3 |  |  |  |  |  |
| 433 | rs4837613 | c | g | 0.4887 | -0.02 | 0.002 | 3.15E-29 | 90.0 |  |  |  |  |  |
| 434 | rs10818576 | t | g | 0.7608 | -0.02 | 0.002 | 4.59E-13 | 65.6 |  |  |  |  |  |
| 435 | rs4836544 | t | c | 0.8846 | 0.02 | 0.003 | 4.26E-09 | 36.8 |  |  |  |  |  |
| 436 | rs62578126 | t | c | 0.3721 | -0.01 | 0.002 | 1.60E-11 | 21.0 |  |  |  |  |  |
| 437 | rs3802342 | c | g | 0.648 | 0.01 | 0.002 | 1.37E-13 | 20.5 |  |  |  |  |  |
| 438 | rs8176672 | t | c | 0.0627 | -0.03 | 0.004 | 7.90E-16 | 47.6 |  |  |  |  |  |
| 439 | rs13300181 | a | g | 0.6455 | 0.01 | 0.002 | 7.53E-11 | 20.6 |  |  |  |  |  |
| 440 | rs141147400 | c | g | 0.2921 | 0.02 | 0.002 | 1.23E-15 | 74.5 |  |  |  |  |  |
| 441 | rs10858246 | c | g | 0.3183 | -0.02 | 0.002 | 2.35E-20 | 78.2 |  |  |  |  |  |
| 442 | rs7020201 | c | g | 0.543 | 0.01 | 0.002 | 3.49E-11 | 22.3 |  |  |  |  |  |
| 443 | rs72761015 | t | c | 0.125 | -0.02 | 0.003 | 2.76E-14 | 39.4 |  |  |  |  |  |
| 444 | rs35288270 | t | c | 0.8656 | 0.03 | 0.003 | 3.44E-32 | 94.3 |  |  |  |  |  |
| 445 | rs35092545 | t | g | 0.2896 | -0.01 | 0.002 | 6.00E-12 | 18.5 |  |  |  |  |  |
| 446 | rs7087801 | t | c | 0.2979 | -0.02 | 0.002 | 7.55E-14 | 75.3 |  |  |  |  |  |
| 447 | rs11014285 | a | g | 0.1654 | 0.03 | 0.003 | 2.89E-40 | 111.9 |  |  |  |  |  |
| 448 | rs2808290 | t | c | 0.5029 | 0.02 | 0.002 | 2.19E-21 | 90.1 |  |  |  |  |  |
| 449 | rs1778871 | c | g | 0.8811 | 0.02 | 0.003 | 6.04E-11 | 37.7 |  |  |  |  |  |
| 450 | rs2490302 | a | t | 0.9137 | 0.02 | 0.003 | 6.24E-11 | 28.4 |  |  |  |  |  |
| 451 | rs10776560 | t | c | 0.4995 | -0.02 | 0.002 | 7.88E-17 | 90.1 |  |  |  |  |  |
| 452 | rs10822055 | a | t | 0.2349 | -0.02 | 0.002 | 2.44E-15 | 64.7 |  |  |  |  |  |
| 453 | rs61849823 | t | c | 0.8357 | -0.02 | 0.003 | 3.16E-14 | 49.5 |  |  |  |  |  |
| 454 | rs77974561 | a | c | 0.0546 | -0.03 | 0.004 | 1.52E-11 | 41.8 |  |  |  |  |  |
| 455 | rs12218358 | c | g | 0.5639 | 0.01 | 0.002 | 6.49E-10 | 22.1 |  |  |  |  |  |
| 456 | rs1171614 | t | c | 0.2309 | 0.02 | 0.002 | 1.13E-13 | 64.0 |  |  |  |  |  |
| 457 | rs10761494 | a | t | 0.3617 | -0.02 | 0.002 | 3.06E-14 | 83.2 |  |  |  |  |  |
| 458 | rs67527161 | t | c | 0.791 | 0.02 | 0.002 | 5.79E-15 | 59.6 |  |  |  |  |  |
| 459 | rs224043 | t | g | 0.5677 | -0.02 | 0.002 | 1.42E-17 | 88.4 |  |  |  |  |  |
| 460 | rs117880827 | t | c | 0.9176 | 0.02 | 0.003 | 1.57E-09 | 27.2 |  |  |  |  |  |
| 461 | rs7916821 | a | g | 0.4945 | 0.02 | 0.002 | 3.79E-21 | 90.1 |  |  |  |  |  |
| 462 | rs7095472 | a | g | 0.4665 | -0.03 | 0.002 | 7.66E-45 | 201.8 |  |  |  |  |  |
| 463 | rs68049170 | a | g | 0.2764 | -0.03 | 0.002 | 2.69E-34 | 162.1 |  |  |  |  |  |
| 464 | rs117543413 | t | c | 0.0176 | -0.07 | 0.007 | 2.47E-21 | 76.3 |  |  |  |  |  |
| 465 | rs10824747 | t | c | 0.4994 | 0.02 | 0.002 | 5.28E-19 | 90.1 |  |  | Outlier |  |  |
| 466 | rs7910211 | t | c | 0.8413 | -0.02 | 0.002 | 1.21E-11 | 48.1 |  |  |  |  |  |
| 467 | rs4934377 | a | t | 0.7297 | -0.02 | 0.002 | 6.65E-16 | 71.1 |  |  |  |  |  |
| 468 | rs2648725 | a | t | 0.2134 | 0.02 | 0.002 | 8.20E-13 | 60.5 |  |  |  |  |  |
| 469 | rs4917612 | c | g | 0.8665 | -0.03 | 0.003 | 4.77E-33 | 93.8 |  |  |  |  |  |
| 470 | rs573605538 | a | t | 0.4182 | 0.01 | 0.002 | 1.76E-14 | 21.9 | Excluded | Excluded | Excluded | Excluded | Excluded |
| 471 | rs1247121 | a | g | 0.4337 | 0.02 | 0.002 | 2.10E-20 | 88.5 |  |  |  |  |  |
| 472 | rs291979 | a | g | 0.2289 | 0.02 | 0.002 | 6.76E-27 | 63.6 |  |  |  |  |  |
| 473 | rs10437498 | a | g | 0.6446 | -0.01 | 0.002 | 3.30E-10 | 20.6 | Excluded | Excluded | Excluded | Excluded |  |
| 474 | rs75356180 | a | t | 0.8802 | 0.02 | 0.003 | 1.35E-10 | 38.0 |  |  |  |  |  |
| 475 | rs4752689 | a | g | 0.5839 | 0.02 | 0.002 | 1.36E-26 | 87.5 |  |  |  |  |  |
| 476 | rs28445838 | a | g | 0.6075 | 0.01 | 0.002 | 2.92E-11 | 21.5 |  |  |  |  |  |
| 477 | rs4962504 | a | g | 0.1162 | -0.02 | 0.003 | 2.13E-10 | 37.0 |  |  |  |  |  |
| 478 | rs372532055 | a | c | 0.3664 | 0.02 | 0.002 | 1.12E-18 | 83.6 | rs1556659 | rs1556659 | rs1556659 | rs1556659 | rs1556659 |
| 479 | rs2734498 | t | c | 0.5798 | 0.01 | 0.002 | 4.51E-15 | 21.9 |  |  |  |  |  |
| 480 | rs35506085 | a | g | 0.1847 | -0.03 | 0.003 | 7.13E-35 | 122.1 |  |  |  |  |  |
| 481 | rs74501871 | a | g | 0.9063 | 0.03 | 0.003 | 1.63E-25 | 68.8 |  |  |  |  |  |
| 482 | rs12271773 | a | g | 0.1159 | 0.03 | 0.003 | 3.44E-17 | 83.1 |  |  |  |  |  |
| 483 | rs143840904 | t | c | 0.0197 | -0.08 | 0.007 | 1.46E-30 | 111.3 |  |  |  |  |  |
| 484 | rs4910048 | t | c | 0.5049 | 0.02 | 0.002 | 1.45E-15 | 90.1 |  |  |  |  |  |
| 485 | rs11042717 | t | c | 0.5104 | 0.03 | 0.002 | 4.04E-53 | 202.6 |  |  |  |  |  |
| 486 | rs12226039 | t | c | 0.5724 | 0.02 | 0.002 | 1.65E-14 | 88.2 | Excluded | Excluded | Excluded | Excluded | Excluded |
| 487 | rs77889556 | a | g | 0.1674 | 0.02 | 0.003 | 1.40E-16 | 50.2 |  |  |  |  |  |
| 488 | rs10832963 | t | g | 0.2555 | 0.02 | 0.002 | 9.23E-21 | 68.5 |  |  |  |  |  |
| 489 | rs117130980 | t | c | 0.9872 | 0.06 | 0.009 | 1.13E-10 | 41.0 |  |  |  |  |  |
| 490 | rs7396827 | t | c | 0.464 | 0.01 | 0.002 | 1.12E-12 | 22.4 |  |  |  |  |  |
| 491 | rs704660 | t | c | 0.4095 | 0.02 | 0.002 | 2.28E-15 | 87.1 |  |  |  |  |  |
| 492 | rs148683230 | a | g | 0.149 | 0.02 | 0.003 | 3.71E-12 | 45.7 |  |  |  |  |  |
| 493 | rs113542381 | a | g | 0.1461 | -0.02 | 0.003 | 2.07E-11 | 44.9 |  |  |  |  |  |
| 494 | rs1164660 | t | g | 0.5825 | -0.02 | 0.002 | 5.17E-15 | 87.6 |  |  |  |  |  |
| 495 | rs117292441 | t | g | 0.0125 | 0.05 | 0.009 | 3.19E-10 | 27.8 |  |  |  |  |  |
| 496 | rs28456 | a | g | 0.6863 | 0.01 | 0.002 | 3.76E-12 | 19.4 |  |  |  |  |  |
| 497 | rs7122950 | t | c | 0.2886 | 0.01 | 0.002 | 7.70E-10 | 18.5 |  |  |  |  |  |
| 498 | rs601310 | t | c | 0.7513 | 0.01 | 0.002 | 2.79E-10 | 16.8 |  |  |  |  |  |
| 499 | rs12790261 | a | c | 0.0827 | -0.05 | 0.003 | 5.90E-40 | 170.8 |  |  |  |  |  |
| 500 | rs11228182 | a | g | 0.2958 | 0.01 | 0.002 | 1.89E-09 | 18.8 |  |  |  |  |  |
| 501 | rs7129320 | a | g | 0.1661 | -0.04 | 0.003 | 7.29E-53 | 199.7 |  |  |  |  |  |
| 502 | rs28378931 | a | g | 0.8934 | -0.02 | 0.003 | 7.26E-17 | 34.3 |  |  |  |  |  |
| 503 | rs10796820 | t | g | 0.529 | -0.02 | 0.002 | 2.59E-22 | 89.8 |  |  |  |  |  |
| 504 | rs1789167 | a | g | 0.3467 | -0.01 | 0.002 | 3.63E-13 | 20.4 |  |  |  |  |  |
| 505 | rs11233117 | c | g | 0.5465 | 0.02 | 0.002 | 4.66E-23 | 89.3 |  |  |  |  |  |
| 506 | rs1876 | t | c | 0.8892 | 0.03 | 0.003 | 6.85E-21 | 79.9 |  |  |  |  |  |
| 507 | rs7902 | a | g | 0.5531 | -0.01 | 0.002 | 5.22E-15 | 22.3 |  |  |  |  |  |
| 508 | rs604723 | t | c | 0.2752 | 0.02 | 0.002 | 8.16E-15 | 71.9 |  |  |  |  |  |
| 509 | rs11217863 | a | g | 0.1162 | -0.03 | 0.003 | 1.06E-19 | 83.2 |  |  |  |  |  |
| 510 | rs12222197 | t | g | 0.5812 | 0.01 | 0.002 | 7.29E-11 | 21.9 |  |  |  |  |  |
| 511 | rs7137546 | a | t | 0.5751 | -0.01 | 0.002 | 9.71E-14 | 22.0 |  |  |  |  |  |
| 512 | rs2159102 | a | t | 0.3956 | -0.01 | 0.002 | 3.57E-10 | 21.5 |  |  |  |  |  |
| 513 | rs35244030 | a | g | 0.1648 | 0.02 | 0.003 | 7.50E-22 | 49.6 |  |  |  |  |  |
| 514 | rs67551338 | t | c | 0.0615 | 0.06 | 0.004 | 2.09E-55 | 187.2 |  |  |  |  |  |
| 515 | rs76895963 | t | g | 0.9793 | -0.16 | 0.007 | 8.23E-112 | 467.8 |  |  |  |  |  |
| 516 | rs10845408 | t | c | 0.3538 | 0.03 | 0.002 | 3.25E-38 | 185.4 |  |  |  |  |  |
| 517 | rs2066827 | t | g | 0.7675 | 0.02 | 0.002 | 1.76E-16 | 64.3 |  |  |  |  |  |
| 518 | rs73069098 | a | g | 0.9308 | 0.02 | 0.004 | 4.28E-10 | 23.2 |  |  |  |  |  |
| 519 | rs10770654 | a | g | 0.3098 | -0.02 | 0.002 | 6.40E-32 | 77.0 |  |  |  |  |  |
| 520 | rs61415458 | t | c | 0.8668 | -0.02 | 0.003 | 1.00E-15 | 41.6 |  |  |  |  |  |
| 521 | rs61688134 | t | c | 0.0143 | -0.06 | 0.008 | 7.88E-15 | 45.7 |  |  |  |  |  |
| 522 | rs11047225 | t | c | 0.4691 | -0.01 | 0.002 | 4.30E-10 | 22.4 |  |  |  |  |  |
| 523 | rs11049576 | t | c | 0.7072 | 0.02 | 0.002 | 9.49E-20 | 74.6 |  |  |  |  |  |
| 524 | rs12831751 | a | c | 0.7143 | -0.02 | 0.002 | 1.57E-16 | 73.5 |  |  |  |  |  |
| 525 | rs6582398 | t | c | 0.6002 | 0.01 | 0.002 | 1.14E-12 | 21.6 |  |  |  |  |  |
| 526 | rs12828089 | a | c | 0.15 | -0.02 | 0.003 | 3.58E-11 | 45.9 |  |  |  |  |  |
| 527 | rs74082087 | a | g | 0.9125 | -0.02 | 0.003 | 1.80E-10 | 28.8 |  |  |  |  |  |
| 528 | rs74385174 | a | g | 0.9808 | 0.07 | 0.007 | 1.54E-25 | 83.1 |  |  |  |  |  |
| 529 | rs12099669 | a | g | 0.6962 | 0.03 | 0.002 | 1.39E-58 | 171.5 |  |  |  |  |  |
| 530 | rs73101415 | a | g | 0.7464 | -0.01 | 0.002 | 2.06E-11 | 17.0 |  |  |  |  |  |
| 531 | rs1990028 | a | g | 0.7467 | 0.02 | 0.002 | 1.06E-12 | 68.1 |  |  |  |  |  |
| 532 | rs61941109 | a | g | 0.2846 | -0.01 | 0.002 | 8.56E-12 | 18.3 |  |  |  |  |  |
| 533 | rs12309572 | t | c | 0.1667 | -0.02 | 0.003 | 4.57E-13 | 50.0 |  |  |  |  |  |
| 534 | rs2071450 | t | c | 0.3675 | -0.02 | 0.002 | 8.85E-19 | 83.7 |  |  |  |  |  |
| 535 | rs17118403 | t | c | 0.0667 | 0.03 | 0.004 | 4.11E-19 | 50.5 |  |  |  |  |  |
| 536 | rs2277339 | t | g | 0.8961 | 0.04 | 0.003 | 2.00E-30 | 134.2 |  |  |  |  |  |
| 537 | rs11172113 | t | c | 0.5877 | 0.02 | 0.002 | 4.57E-17 | 87.3 |  |  |  |  |  |
| 538 | rs17178006 | t | g | 0.885 | 0.02 | 0.003 | 2.27E-11 | 36.7 |  |  |  |  |  |
| 539 | rs10878339 | a | g | 0.9349 | 0.04 | 0.004 | 1.83E-23 | 87.7 |  |  |  |  |  |
| 540 | rs10748128 | t | g | 0.3446 | 0.03 | 0.002 | 6.77E-38 | 183.1 |  |  |  |  |  |
| 541 | rs79237130 | a | t | 0.1606 | -0.02 | 0.003 | 1.08E-10 | 48.6 |  |  |  |  |  |
| 542 | rs61754233 | c | g | 0.02 | 0.04 | 0.007 | 2.36E-10 | 28.2 |  |  |  |  |  |
| 543 | rs7976940 | a | g | 0.9323 | 0.03 | 0.004 | 9.02E-14 | 51.2 |  |  |  |  |  |
| 544 | rs7980592 | a | g | 0.2929 | -0.02 | 0.002 | 1.46E-13 | 74.6 |  |  |  |  |  |
| 545 | rs9634212 | a | c | 0.221 | 0.05 | 0.002 | 8.59E-95 | 387.9 |  |  |  |  |  |
| 546 | rs7953280 | c | g | 0.5071 | -0.03 | 0.002 | 3.20E-64 | 202.7 |  |  |  |  |  |
| 547 | rs4399366 | t | c | 0.2829 | -0.02 | 0.002 | 7.03E-13 | 73.1 |  |  |  |  |  |
| 548 | rs7971536 | a | t | 0.4945 | -0.02 | 0.002 | 1.07E-24 | 90.1 |  |  |  |  |  |
| 549 | rs66500550 | c | g | 0.9579 | 0.04 | 0.005 | 2.10E-17 | 58.1 |  |  |  |  |  |
| 550 | rs35762 | t | c | 0.1525 | 0.02 | 0.003 | 4.25E-17 | 46.6 |  |  |  |  |  |
| 551 | rs2647873 | a | g | 0.518 | 0.02 | 0.002 | 3.21E-23 | 89.9 |  |  |  |  |  |
| 552 | rs117441946 | a | t | 0.0488 | -0.03 | 0.004 | 9.11E-10 | 37.6 |  |  |  |  |  |
| 553 | rs3782709 | a | g | 0.6442 | 0.01 | 0.002 | 7.53E-11 | 20.6 |  |  |  |  |  |
| 554 | rs3764002 | t | c | 0.2617 | 0.03 | 0.002 | 4.47E-39 | 156.6 |  |  |  |  |  |
| 555 | rs1541597 | t | g | 0.2012 | 0.01 | 0.002 | 4.91E-10 | 14.5 |  |  |  |  |  |
| 556 | rs3184504 | t | c | 0.483 | -0.02 | 0.002 | 2.71E-22 | 90.0 | Outlier | Outlier | Outlier |  |  |
| 557 | rs1061657 | t | c | 0.7421 | -0.02 | 0.002 | 3.53E-16 | 68.9 |  |  |  |  |  |
| 558 | rs7305948 | t | c | 0.1459 | -0.02 | 0.003 | 5.11E-20 | 44.9 |  |  |  |  |  |
| 559 | rs555994863 | t | c | 0.0191 | 0.04 | 0.007 | 3.85E-09 | 27.0 | Excluded | Excluded | Excluded | Excluded | Excluded |
| 560 | rs7969643 | t | c | 0.2524 | -0.01 | 0.002 | 8.75E-11 | 17.0 |  |  |  |  |  |
| 561 | rs28576953 | t | c | 0.2048 | 0.03 | 0.002 | 5.97E-38 | 132.0 |  |  |  |  |  |
| 562 | rs7301953 | a | g | 0.3122 | -0.02 | 0.002 | 3.86E-20 | 77.4 |  |  |  |  |  |
| 563 | rs2229840 | t | c | 0.1597 | 0.03 | 0.003 | 3.02E-40 | 108.8 |  |  |  |  |  |
| 564 | rs885389 | a | g | 0.3328 | 0.03 | 0.002 | 6.37E-37 | 180.0 |  |  |  |  |  |
| 565 | rs6598200 | a | g | 0.1586 | 0.02 | 0.003 | 8.16E-14 | 48.1 |  |  |  |  |  |
| 566 | rs7321635 | a | c | 0.3535 | 0.01 | 0.002 | 2.58E-11 | 20.6 |  |  |  |  |  |
| 567 | rs61944841 | a | g | 0.4138 | 0.03 | 0.002 | 3.54E-37 | 196.7 |  |  |  |  |  |
| 568 | rs4769763 | c | g | 0.6076 | -0.02 | 0.002 | 2.75E-16 | 85.9 |  |  |  |  |  |
| 569 | rs17532490 | a | g | 0.0854 | -0.02 | 0.003 | 7.45E-10 | 28.1 |  |  |  |  |  |
| 570 | rs9594689 | t | g | 0.6261 | 0.01 | 0.002 | 1.19E-12 | 21.1 | Excluded | Excluded | Excluded | Excluded | Excluded |
| 571 | rs9596092 | a | t | 0.9139 | 0.02 | 0.004 | 7.80E-12 | 28.3 |  |  |  |  |  |
| 572 | rs9591310 | t | c | 0.1994 | -0.02 | 0.002 | 1.09E-29 | 57.5 |  |  |  |  |  |
| 573 | rs56273425 | a | c | 0.0255 | -0.04 | 0.006 | 3.71E-10 | 35.8 |  |  |  |  |  |
| 574 | rs3116602 | t | g | 0.7847 | 0.06 | 0.002 | 9.52E-155 | 548.3 |  |  |  |  |  |
| 575 | rs4531637 | a | c | 0.853 | -0.02 | 0.003 | 1.46E-12 | 45.2 | Excluded | Excluded | Excluded | Excluded | Excluded |
| 576 | rs17074618 | t | c | 0.1535 | 0.03 | 0.003 | 1.82E-24 | 105.3 |  |  |  |  |  |
| 577 | rs7328187 | t | g | 0.5024 | -0.01 | 0.002 | 1.19E-09 | 22.5 |  |  |  |  |  |
| 578 | rs7319334 | a | g | 0.5194 | -0.01 | 0.002 | 3.71E-09 | 22.5 |  |  |  |  |  |
| 579 | rs3818416 | a | c | 0.2355 | -0.03 | 0.002 | 2.01E-35 | 146.0 |  |  |  |  |  |
| 580 | rs9515919 | a | t | 0.3693 | 0.02 | 0.002 | 6.01E-16 | 83.9 |  |  |  |  |  |
| 581 | rs9517483 | a | g | 0.301 | 0.02 | 0.002 | 2.26E-18 | 75.8 |  |  |  |  |  |
| 582 | rs7323466 | t | c | 0.3488 | -0.02 | 0.002 | 5.91E-14 | 81.8 |  |  |  |  |  |
| 583 | rs78525785 | t | c | 0.62 | -0.02 | 0.002 | 9.81E-18 | 84.9 | Excluded | Excluded | Excluded | Excluded | Excluded |
| 584 | rs8019890 | a | c | 0.5313 | 0.03 | 0.002 | 1.96E-38 | 201.9 |  |  |  |  |  |
| 585 | rs28663442 | t | c | 0.1761 | 0.02 | 0.003 | 1.11E-19 | 52.3 |  |  |  |  |  |
| 586 | rs1004030 | t | c | 0.5751 | 0.01 | 0.002 | 2.34E-10 | 22.0 |  |  |  |  |  |
| 587 | rs45528934 | t | c | 0.1623 | 0.03 | 0.003 | 1.97E-24 | 110.2 |  |  |  |  |  |
| 588 | rs17184689 | t | g | 0.7452 | -0.01 | 0.002 | 7.76E-10 | 17.1 |  |  |  |  |  |
| 589 | rs36226649 | t | c | 0.9333 | -0.05 | 0.004 | 3.05E-37 | 140.2 |  |  |  |  |  |
| 590 | rs4344657 | c | g | 0.7292 | -0.02 | 0.002 | 7.54E-14 | 71.1 |  |  |  |  |  |
| 591 | rs8904 | a | g | 0.363 | -0.02 | 0.002 | 1.52E-15 | 83.3 |  |  |  |  |  |
| 592 | rs113053299 | a | c | 0.0467 | 0.03 | 0.005 | 4.18E-11 | 36.1 |  |  |  |  |  |
| 593 | rs10483727 | t | c | 0.3891 | 0.04 | 0.002 | 6.73E-80 | 342.7 |  |  |  |  |  |
| 594 | rs7147987 | a | g | 0.5387 | 0.02 | 0.002 | 1.36E-23 | 89.5 |  |  |  |  |  |
| 595 | rs227447 | c | g | 0.6664 | -0.02 | 0.002 | 1.48E-24 | 80.1 |  |  |  |  |  |
| 596 | rs2526919 | a | g | 0.4704 | -0.01 | 0.002 | 5.54E-12 | 22.4 |  |  |  |  |  |
| 597 | rs2070598 | a | g | 0.4559 | 0.02 | 0.002 | 6.36E-27 | 89.4 |  |  |  |  |  |
| 598 | rs1125773 | a | c | 0.48 | 0.02 | 0.002 | 6.29E-16 | 89.9 |  |  |  |  |  |
| 599 | rs2295164 | t | c | 0.4652 | -0.01 | 0.002 | 5.25E-14 | 22.4 |  |  |  |  |  |
| 600 | rs117068593 | t | c | 0.1895 | 0.04 | 0.002 | 8.83E-62 | 221.4 |  |  |  |  |  |
| 601 | rs7156335 | t | c | 0.9088 | -0.03 | 0.003 | 3.63E-18 | 67.2 |  |  |  |  |  |
| 602 | rs28929474 | t | c | 0.0198 | 0.05 | 0.007 | 1.05E-14 | 43.7 |  |  |  |  |  |
| 603 | rs61985998 | t | c | 0.4975 | 0.01 | 0.002 | 2.19E-12 | 22.5 |  |  |  |  |  |
| 604 | rs3783350 | a | g | 0.7967 | -0.02 | 0.002 | 4.88E-11 | 58.3 |  |  |  |  |  |
| 605 | rs1190543 | a | g | 0.2278 | -0.02 | 0.002 | 8.28E-14 | 63.4 |  |  |  |  |  |
| 606 | rs34633205 | t | c | 0.0685 | 0.03 | 0.004 | 3.71E-12 | 51.7 |  |  |  |  |  |
| 607 | rs2174008 | c | g | 0.4975 | -0.02 | 0.002 | 5.89E-24 | 90.1 |  |  |  |  |  |
| 608 | rs548932774 | a | g | 0.3472 | -0.02 | 0.002 | 2.40E-22 | 81.7 | rs111684993 | rs111684993 | rs111684993 | rs111684993 | rs111684993 |
| 609 | rs11855017 | a | c | 0.1817 | 0.02 | 0.003 | 8.85E-14 | 53.6 |  |  |  |  |  |
| 610 | rs62020698 | t | c | 0.0917 | -0.02 | 0.003 | 8.58E-14 | 30.0 |  |  |  |  |  |
| 611 | rs76825670 | a | g | 0.7834 | -0.02 | 0.002 | 4.87E-12 | 61.1 | Excluded | Excluded | Excluded | Excluded | Excluded |
| 612 | rs373736365 | t | g | 0.2843 | -0.02 | 0.002 | 4.53E-18 | 73.3 | Excluded | Excluded | Excluded | Excluded | Excluded |
| 613 | rs1550327 | t | c | 0.6078 | 0.01 | 0.002 | 4.75E-09 | 21.5 |  |  |  |  |  |
| 614 | rs62043796 | t | c | 0.8939 | -0.02 | 0.003 | 1.02E-12 | 34.2 |  |  |  |  |  |
| 615 | rs17205463 | t | c | 0.4477 | -0.03 | 0.002 | 4.21E-43 | 200.5 |  |  |  |  |  |
| 616 | rs72753851 | c | g | 0.7773 | 0.01 | 0.002 | 3.62E-11 | 15.6 |  |  |  |  |  |
| 617 | rs2278604 | a | c | 0.2684 | 0.03 | 0.002 | 6.60E-40 | 159.2 |  |  |  |  |  |
| 618 | rs7179372 | a | g | 0.2063 | -0.01 | 0.002 | 5.57E-11 | 14.7 |  |  |  |  |  |
| 619 | rs35874463 | a | g | 0.9423 | -0.03 | 0.004 | 2.92E-12 | 44.1 |  |  |  |  |  |
| 620 | rs2680338 | a | g | 0.5829 | -0.02 | 0.002 | 9.84E-17 | 87.6 |  |  |  |  |  |
| 621 | rs5742915 | t | c | 0.5395 | -0.02 | 0.002 | 9.33E-39 | 89.5 |  |  |  |  |  |
| 622 | rs7170787 | a | g | 0.2516 | 0.02 | 0.002 | 2.94E-18 | 67.8 |  |  |  |  |  |
| 623 | rs7177344 | t | c | 0.2607 | -0.02 | 0.002 | 2.51E-14 | 69.4 |  |  |  |  |  |
| 624 | rs2202885 | t | c | 0.7057 | 0.01 | 0.002 | 9.41E-10 | 18.7 |  |  |  |  |  |
| 625 | rs11638600 | a | g | 0.3276 | -0.02 | 0.002 | 1.85E-29 | 79.4 |  |  |  |  |  |
| 626 | rs72765638 | c | g | 0.122 | -0.02 | 0.003 | 5.48E-17 | 38.6 |  |  |  |  |  |
| 627 | rs11633371 | t | g | 0.4764 | 0.02 | 0.002 | 1.30E-27 | 89.9 |  |  |  |  |  |
| 628 | rs56238630 | t | c | 0.9708 | 0.07 | 0.006 | 6.58E-39 | 125.1 |  |  |  |  |  |
| 629 | rs34949187 | a | g | 0.1864 | -0.03 | 0.002 | 4.75E-40 | 122.9 |  |  |  |  |  |
| 630 | rs961288 | c | g | 0.4132 | 0.01 | 0.002 | 1.11E-12 | 21.8 |  |  |  |  |  |
| 631 | rs4702 | a | g | 0.5566 | -0.01 | 0.002 | 6.30E-13 | 22.2 |  |  |  |  |  |
| 632 | rs74379684 | t | c | 0.0751 | -0.03 | 0.004 | 4.39E-14 | 56.3 |  |  |  |  |  |
| 633 | rs8035878 | t | c | 0.4523 | 0.01 | 0.002 | 1.17E-12 | 22.3 |  |  |  |  |  |
| 634 | rs7402983 | a | c | 0.3983 | 0.02 | 0.002 | 3.71E-23 | 86.3 |  |  |  |  |  |
| 635 | rs2871865 | c | g | 0.8841 | 0.05 | 0.003 | 3.40E-62 | 230.8 |  |  |  |  |  |
| 636 | rs8035410 | c | g | 0.8351 | 0.03 | 0.003 | 1.76E-23 | 111.6 |  |  |  |  |  |
| 637 | rs3848364 | t | g | 0.7342 | 0.01 | 0.002 | 2.16E-11 | 17.6 |  |  |  |  |  |
| 638 | rs12051245 | t | c | 0.7682 | -0.03 | 0.002 | 2.56E-40 | 144.4 |  |  |  |  |  |
| 639 | rs73483874 | t | c | 0.6888 | -0.02 | 0.002 | 1.05E-14 | 77.2 |  |  |  |  |  |
| 640 | rs548411 | t | g | 0.2455 | -0.02 | 0.002 | 5.93E-17 | 66.7 |  |  |  |  |  |
| 641 | rs2092389 | t | c | 0.2081 | -0.02 | 0.002 | 1.03E-15 | 59.4 |  |  |  |  |  |
| 642 | rs8046527 | c | g | 0.131 | 0.02 | 0.003 | 6.30E-10 | 41.0 |  |  |  |  |  |
| 643 | rs3784828 | t | c | 0.1274 | 0.02 | 0.003 | 2.18E-12 | 40.0 |  |  |  |  |  |
| 644 | rs35816944 | a | g | 0.0066 | -0.11 | 0.011 | 2.49E-23 | 71.4 | Excluded |  | Excluded | Excluded |  |
| 645 | rs116092985 | a | g | 0.9042 | 0.04 | 0.003 | 1.17E-34 | 124.8 |  |  |  |  |  |
| 646 | rs112805476 | a | g | 0.2622 | -0.02 | 0.002 | 1.58E-27 | 69.7 |  |  |  |  |  |
| 647 | rs74367875 | t | c | 0.035 | 0.05 | 0.005 | 1.26E-17 | 76.0 |  |  |  |  |  |
| 648 | rs1045475 | a | g | 0.1782 | -0.02 | 0.003 | 1.95E-19 | 52.8 |  |  |  |  |  |
| 649 | rs2908660 | t | c | 0.5649 | -0.01 | 0.002 | 2.05E-09 | 22.1 |  |  |  |  |  |
| 650 | rs9924441 | t | c | 0.2539 | 0.01 | 0.002 | 2.21E-10 | 17.1 |  |  |  |  |  |
| 651 | rs246177 | t | c | 0.3681 | 0.02 | 0.002 | 2.04E-27 | 83.8 |  |  |  |  |  |
| 652 | rs1136001 | t | g | 0.2965 | -0.02 | 0.002 | 3.02E-16 | 75.1 |  |  |  |  |  |
| 653 | rs9939257 | t | c | 0.5101 | 0.02 | 0.002 | 4.31E-15 | 90.0 | Excluded | Excluded | Excluded | Excluded | Excluded |
| 654 | rs78457529 | t | c | 0.0117 | -0.09 | 0.009 | 1.22E-24 | 84.4 |  |  |  |  |  |
| 655 | rs4788218 | t | c | 0.599 | -0.03 | 0.002 | 5.53E-46 | 194.7 |  |  |  |  |  |
| 656 | rs62033029 | a | g | 0.2064 | -0.01 | 0.002 | 1.73E-09 | 14.8 |  |  |  |  |  |
| 657 | rs72801843 | a | t | 0.3015 | 0.03 | 0.002 | 8.83E-52 | 170.7 |  |  |  |  |  |
| 658 | rs55872725 | t | c | 0.4038 | 0.02 | 0.002 | 1.23E-32 | 86.7 |  |  |  |  |  |
| 659 | rs7203984 | a | c | 0.8067 | -0.02 | 0.002 | 1.85E-12 | 56.2 |  |  |  |  |  |
| 660 | rs35268848 | a | c | 0.0119 | 0.07 | 0.01 | 2.83E-13 | 51.9 |  |  |  | Excluded |  |
| 661 | rs4985445 | a | g | 0.5435 | 0.02 | 0.002 | 3.30E-20 | 89.4 |  |  |  |  |  |
| 662 | rs4788811 | a | g | 0.8589 | 0.02 | 0.003 | 3.14E-12 | 43.7 |  |  |  |  |  |
| 663 | rs543099777 | a | g | 0.1822 | 0.02 | 0.003 | 1.89E-10 | 53.7 | Excluded | Excluded | Excluded | Excluded | Excluded |
| 664 | rs2925979 | t | c | 0.2999 | 0.01 | 0.002 | 1.61E-11 | 18.9 |  |  |  |  |  |
| 665 | rs28377284 | c | g | 0.196 | -0.02 | 0.002 | 7.28E-21 | 56.8 |  |  |  |  |  |
| 666 | rs9319453 | t | g | 0.6671 | -0.01 | 0.002 | 2.39E-09 | 20.0 |  |  |  |  |  |
| 667 | rs56360131 | t | c | 0.1955 | -0.02 | 0.002 | 3.85E-11 | 56.7 |  |  |  |  |  |
| 668 | rs34406692 | t | c | 0.0655 | 0.03 | 0.004 | 6.49E-14 | 49.6 |  |  |  |  |  |
| 669 | rs8054549 | a | c | 0.4486 | -0.03 | 0.002 | 3.37E-39 | 200.6 |  |  |  |  |  |
| 670 | rs112898929 | a | t | 0.0797 | -0.03 | 0.004 | 9.59E-17 | 59.5 |  |  |  |  |  |
| 671 | rs76520574 | t | c | 0.0413 | -0.05 | 0.005 | 4.13E-22 | 89.2 |  |  |  |  |  |
| 672 | rs45580931 | t | c | 0.0428 | -0.03 | 0.005 | 3.83E-11 | 33.2 |  |  |  |  |  |
| 673 | rs2968478 | t | g | 0.4174 | 0.01 | 0.002 | 5.60E-14 | 21.9 |  |  |  |  |  |
| 674 | rs62070319 | t | c | 0.4427 | -0.02 | 0.002 | 2.54E-20 | 88.9 | rs57696383 | rs57696383 | rs57696383 | rs57696383 | rs57696383 |
| 675 | rs2663343 | a | g | 0.3124 | -0.02 | 0.002 | 6.83E-15 | 77.4 |  |  |  |  |  |
| 676 | rs9905106 | t | c | 0.2646 | 0.02 | 0.002 | 7.51E-14 | 70.1 |  |  |  |  |  |
| 677 | rs72820359 | a | g | 0.0733 | -0.02 | 0.004 | 4.22E-11 | 24.5 |  |  |  |  |  |
| 678 | rs413016 | t | c | 0.2642 | 0.02 | 0.002 | 3.79E-16 | 70.0 |  |  |  |  |  |
| 679 | rs57513571 | t | c | 0.2004 | -0.02 | 0.002 | 1.62E-11 | 57.7 |  |  |  |  |  |
| 680 | rs9905997 | a | g | 0.5457 | -0.01 | 0.002 | 6.59E-10 | 22.3 |  |  |  |  |  |
| 681 | rs34914463 | t | c | 0.8687 | -0.04 | 0.003 | 9.81E-40 | 164.4 |  |  |  |  |  |
| 682 | rs78378222 | t | g | 0.9877 | -0.14 | 0.009 | 4.51E-56 | 214.5 |  |  |  |  |  |
| 683 | rs7223668 | a | g | 0.4712 | -0.01 | 0.002 | 2.24E-11 | 22.4 |  |  |  |  |  |
| 684 | rs1242507 | a | g | 0.5613 | 0.01 | 0.002 | 9.71E-12 | 22.2 |  |  |  |  |  |
| 685 | rs7213608 | t | c | 0.675 | 0.03 | 0.002 | 9.63E-39 | 177.9 |  |  |  |  |  |
| 686 | rs11545700 | a | c | 0.1251 | -0.02 | 0.003 | 7.84E-18 | 39.4 |  |  |  |  |  |
| 687 | rs74411146 | a | g | 0.9575 | -0.03 | 0.005 | 3.59E-10 | 33.0 |  |  |  |  |  |
| 688 | rs72823964 | t | c | 0.1321 | 0.03 | 0.003 | 1.07E-24 | 92.9 |  |  |  |  |  |
| 689 | rs57812147 | a | g | 0.0659 | -0.04 | 0.004 | 1.77E-20 | 88.7 |  |  |  |  |  |
| 690 | rs6505216 | t | g | 0.233 | -0.05 | 0.002 | 1.83E-101 | 402.7 |  |  |  |  |  |
| 691 | rs2854332 | a | t | 0.6993 | -0.02 | 0.002 | 1.58E-25 | 75.8 |  |  |  |  |  |
| 692 | rs2338115 | t | c | 0.5555 | 0.03 | 0.002 | 1.04E-39 | 200.2 |  |  |  |  |  |
| 693 | rs190471737 | a | g | 0.9332 | -0.02 | 0.004 | 4.40E-10 | 22.5 |  |  |  |  |  |
| 694 | rs8073219 | a | g | 0.2564 | 0.02 | 0.002 | 2.52E-12 | 68.7 |  |  |  |  |  |
| 695 | rs12603963 | t | c | 0.6303 | -0.01 | 0.002 | 3.72E-09 | 21.0 |  |  |  |  |  |
| 696 | rs45445495 | a | c | 0.0495 | -0.04 | 0.005 | 1.20E-18 | 67.8 |  |  |  |  |  |
| 697 | rs11867858 | t | c | 0.2076 | 0.02 | 0.002 | 1.53E-11 | 59.3 |  |  |  |  |  |
| 698 | rs197907 | c | g | 0.2778 | -0.01 | 0.002 | 2.07E-10 | 18.1 |  |  |  |  |  |
| 699 | rs118127175 | t | g | 0.0372 | -0.04 | 0.005 | 1.76E-17 | 51.6 | Outlier |  |  |  |  |
| 700 | rs28613067 | a | g | 0.2768 | 0.02 | 0.002 | 1.30E-26 | 72.1 |  |  |  |  |  |
| 701 | rs78270829 | t | g | 0.982 | -0.05 | 0.007 | 1.56E-10 | 39.8 |  |  |  |  |  |
| 702 | rs4794005 | a | g | 0.4444 | 0.02 | 0.002 | 8.84E-15 | 89.0 |  |  |  |  |  |
| 703 | rs9303375 | a | g | 0.0597 | 0.03 | 0.004 | 1.46E-13 | 45.5 |  |  |  |  |  |
| 704 | rs72829750 | t | c | 0.4001 | -0.02 | 0.002 | 2.15E-19 | 86.5 |  |  |  |  |  |
| 705 | rs73991579 | a | g | 0.9133 | -0.02 | 0.003 | 6.09E-14 | 28.5 |  |  |  |  |  |
| 706 | rs8073455 | t | c | 0.4941 | -0.01 | 0.002 | 1.91E-11 | 22.5 |  |  |  |  |  |
| 707 | rs56251713 | a | t | 0.6235 | -0.01 | 0.002 | 1.13E-11 | 21.1 | Excluded | Excluded | Excluded | Excluded | Excluded |
| 708 | rs9905385 | a | g | 0.3293 | 0.03 | 0.002 | 1.94E-63 | 179.1 |  |  |  |  |  |
| 709 | rs2079796 | c | g | 0.3702 | 0.02 | 0.002 | 7.30E-21 | 84.0 |  |  |  |  |  |
| 710 | rs11657101 | a | g | 0.3612 | 0.02 | 0.002 | 3.42E-16 | 83.1 |  |  |  |  |  |
| 711 | rs78119823 | a | t | 0.0459 | -0.03 | 0.005 | 4.70E-11 | 35.5 |  |  |  |  |  |
| 712 | rs2005172 | a | c | 0.3603 | -0.05 | 0.002 | 2.35E-128 | 519.5 |  |  |  |  |  |
| 713 | rs11568828 | t | c | 0.8826 | 0.06 | 0.003 | 8.06E-81 | 336.2 |  |  |  |  |  |
| 714 | rs7225219 | a | t | 0.6584 | -0.01 | 0.002 | 1.51E-12 | 20.3 |  |  |  |  |  |
| 715 | rs2676298 | t | c | 0.853 | -0.03 | 0.003 | 2.39E-23 | 101.6 |  |  |  |  |  |
| 716 | rs12452505 | c | g | 0.8578 | 0.03 | 0.003 | 6.61E-23 | 98.9 |  |  |  |  |  |
| 717 | rs77542162 | a | g | 0.9774 | -0.06 | 0.006 | 1.49E-19 | 71.6 |  |  |  |  |  |
| 718 | rs12451722 | t | c | 0.585 | -0.02 | 0.002 | 1.35E-21 | 87.5 |  |  |  |  |  |
| 719 | rs173135 | t | c | 0.115 | -0.03 | 0.003 | 3.25E-30 | 82.5 |  |  |  |  |  |
| 720 | rs236532 | t | c | 0.6077 | -0.02 | 0.002 | 2.26E-16 | 85.9 |  |  |  |  |  |
| 721 | rs9909320 | t | g | 0.4422 | -0.02 | 0.002 | 2.25E-16 | 88.9 |  |  |  |  |  |
| 722 | rs820212 | t | c | 0.3748 | -0.01 | 0.002 | 2.54E-10 | 21.1 |  |  |  |  |  |
| 723 | rs36000545 | a | g | 0.6043 | 0.02 | 0.002 | 2.56E-29 | 86.1 |  |  |  |  |  |
| 724 | rs28507130 | a | t | 0.3657 | 0.02 | 0.002 | 7.16E-23 | 83.6 |  |  |  |  |  |
| 725 | rs4969474 | a | g | 0.4187 | -0.01 | 0.002 | 1.27E-10 | 21.9 |  |  |  |  |  |
| 726 | rs12602034 | t | c | 0.6353 | -0.02 | 0.002 | 4.28E-19 | 83.5 |  |  |  |  |  |
| 727 | rs4121583 | t | c | 0.6192 | 0.01 | 0.002 | 4.62E-09 | 21.2 |  |  |  |  |  |
| 728 | rs1786263 | t | g | 0.6055 | -0.02 | 0.002 | 1.03E-22 | 86.1 |  |  |  |  |  |
| 729 | rs72882867 | t | c | 0.0429 | -0.03 | 0.005 | 1.26E-12 | 33.3 |  |  |  |  |  |
| 730 | rs113232639 | a | g | 0.493 | 0.03 | 0.002 | 4.79E-64 | 202.7 |  |  |  |  |  |
| 731 | rs9957318 | a | g | 0.652 | -0.02 | 0.002 | 1.02E-20 | 81.7 |  |  |  |  |  |
| 732 | rs12962050 | a | g | 0.6445 | 0.02 | 0.002 | 1.52E-14 | 82.5 |  |  |  |  |  |
| 733 | rs1460510 | t | c | 0.2748 | 0.01 | 0.002 | 1.34E-09 | 17.9 |  |  |  |  |  |
| 734 | rs111219378 | t | c | 0.1289 | -0.02 | 0.003 | 2.32E-11 | 40.4 | Excluded | Excluded | Excluded | Excluded | Excluded |
| 735 | rs7229520 | a | g | 0.6616 | -0.02 | 0.002 | 2.57E-34 | 80.7 |  |  |  |  |  |
| 736 | rs33973388 | t | g | 0.4353 | 0.02 | 0.002 | 1.45E-38 | 88.6 |  |  |  |  |  |
| 737 | rs73441521 | t | g | 0.9489 | 0.02 | 0.004 | 1.98E-09 | 17.5 |  |  |  |  |  |
| 738 | rs17065909 | t | c | 0.176 | -0.02 | 0.003 | 1.41E-15 | 52.2 |  |  |  |  |  |
| 739 | rs74494415 | t | c | 0.0398 | -0.04 | 0.005 | 1.82E-17 | 55.1 |  |  |  |  |  |
| 740 | rs60389750 | t | c | 0.3139 | -0.02 | 0.002 | 1.06E-16 | 77.6 |  |  |  |  |  |
| 741 | rs8112948 | a | t | 0.2856 | -0.03 | 0.002 | 4.24E-42 | 165.4 | Excluded | Excluded | Excluded | Excluded | Excluded |
| 742 | rs12610084 | a | g | 0.2017 | 0.02 | 0.002 | 1.22E-13 | 58.0 |  |  |  |  |  |
| 743 | rs56208656 | a | g | 0.1103 | -0.03 | 0.003 | 2.99E-17 | 79.5 |  |  |  |  |  |
| 744 | rs732716 | a | g | 0.7038 | -0.02 | 0.002 | 5.82E-16 | 75.1 |  | Outlier |  |  |  |
| 745 | rs8887 | t | c | 0.4284 | 0.01 | 0.002 | 8.09E-12 | 22.1 |  |  |  |  |  |
| 746 | rs7508325 | a | g | 0.1839 | -0.02 | 0.002 | 2.16E-13 | 54.1 |  |  |  |  |  |
| 747 | rs2602713 | a | c | 0.5603 | -0.02 | 0.002 | 1.68E-29 | 88.8 |  |  |  |  |  |
| 748 | rs1864193 | a | c | 0.1416 | -0.02 | 0.003 | 4.79E-16 | 43.8 |  |  |  |  |  |
| 749 | rs8107967 | a | g | 0.4341 | 0.02 | 0.002 | 9.52E-20 | 88.5 |  |  |  |  |  |
| 750 | rs35279560 | t | g | 0.3091 | 0.02 | 0.002 | 1.01E-13 | 76.9 |  |  |  |  |  |
| 751 | rs62621197 | t | c | 0.0372 | -0.04 | 0.005 | 1.11E-17 | 51.6 |  |  |  |  |  |
| 752 | rs12979274 | t | c | 0.4845 | -0.02 | 0.002 | 3.03E-16 | 90.0 |  |  |  |  |  |
| 753 | rs8104479 | a | g | 0.1506 | -0.02 | 0.003 | 8.85E-12 | 46.1 |  |  |  |  |  |
| 754 | rs10948 | t | g | 0.6638 | -0.03 | 0.002 | 3.43E-36 | 180.9 |  |  |  |  |  |
| 755 | rs2421206 | t | g | 0.6567 | 0.02 | 0.002 | 3.86E-18 | 81.2 |  |  |  |  |  |
| 756 | rs1042164 | t | c | 0.1837 | -0.02 | 0.002 | 2.11E-12 | 54.0 |  |  |  |  |  |
| 757 | rs7246865 | a | g | 0.2606 | -0.02 | 0.002 | 9.89E-22 | 69.4 |  |  |  |  |  |
| 758 | rs7260450 | t | g | 0.3723 | -0.01 | 0.002 | 1.01E-12 | 21.0 |  |  |  |  |  |
| 759 | rs34647936 | t | g | 0.8146 | 0.02 | 0.002 | 3.61E-24 | 54.4 |  |  |  |  |  |
| 760 | rs2287821 | t | c | 0.5091 | -0.02 | 0.002 | 6.95E-16 | 90.0 |  |  |  |  |  |
| 761 | rs28616221 | a | g | 0.1794 | -0.02 | 0.003 | 5.59E-14 | 53.0 |  |  |  |  |  |
| 762 | rs7249000 | c | g | 0.8363 | -0.02 | 0.003 | 2.06E-09 | 49.3 |  |  |  |  |  |
| 763 | rs62108897 | a | c | 0.5246 | -0.02 | 0.002 | 7.73E-19 | 89.8 |  |  |  |  |  |
| 764 | rs34934920 | t | c | 0.025 | -0.04 | 0.006 | 4.14E-09 | 35.1 |  |  |  |  |  |
| 765 | rs304724 | t | c | 0.5322 | 0.01 | 0.002 | 1.30E-10 | 22.4 |  |  |  |  |  |
| 766 | rs8105903 | a | c | 0.5496 | 0.01 | 0.002 | 2.02E-12 | 22.3 |  |  |  |  |  |
| 767 | rs45474992 | t | c | 0.0362 | -0.06 | 0.005 | 2.17E-33 | 113.1 |  |  |  |  |  |
| 768 | rs12986064 | t | c | 0.4889 | -0.01 | 0.002 | 4.31E-14 | 22.5 | Excluded | Excluded | Excluded | Excluded | Excluded |
| 769 | rs4252548 | t | c | 0.0218 | -0.08 | 0.006 | 4.84E-35 | 122.9 |  |  |  |  |  |
| 770 | rs147110934 | t | g | 0.0243 | -0.07 | 0.006 | 9.39E-32 | 104.6 |  |  |  |  |  |
| 771 | rs55706256 | t | g | 0.3783 | 0.01 | 0.002 | 8.90E-13 | 21.2 |  |  |  |  |  |
| 772 | rs2236096 | t | c | 0.7673 | -0.02 | 0.002 | 1.29E-15 | 64.3 |  |  |  |  |  |
| 773 | rs1741344 | t | c | 0.6345 | -0.01 | 0.002 | 4.87E-12 | 20.9 |  |  |  |  |  |
| 774 | rs6054390 | a | g | 0.628 | -0.02 | 0.002 | 1.45E-21 | 84.2 |  |  |  |  |  |
| 775 | rs2650965 | a | g | 0.6707 | 0.01 | 0.002 | 6.34E-14 | 19.9 |  |  |  |  |  |
| 776 | rs4815952 | t | c | 0.4779 | 0.02 | 0.002 | 1.69E-17 | 89.9 |  |  |  |  |  |
| 777 | rs6117726 | t | c | 0.5064 | -0.01 | 0.002 | 7.44E-14 | 22.5 |  |  |  |  |  |
| 778 | rs889509 | a | g | 0.5827 | 0.01 | 0.002 | 2.42E-12 | 21.9 |  |  |  |  |  |
| 779 | rs73125634 | t | g | 0.2782 | -0.02 | 0.002 | 5.11E-20 | 72.3 |  |  |  |  |  |
| 780 | rs76657242 | t | c | 0.0627 | -0.03 | 0.004 | 4.98E-12 | 47.6 |  |  |  |  |  |
| 781 | rs6082354 | a | c | 0.3325 | 0.02 | 0.002 | 1.19E-32 | 80.0 |  |  |  |  |  |
| 782 | rs74397018 | a | g | 0.0431 | -0.03 | 0.005 | 1.18E-11 | 33.4 |  |  |  |  |  |
| 783 | rs910085 | t | g | 0.6128 | 0.02 | 0.002 | 2.88E-19 | 85.5 |  |  |  |  |  |
| 784 | rs34879158 | a | c | 0.737 | 0.04 | 0.002 | 1.55E-63 | 279.4 |  |  |  |  |  |
| 785 | rs143384 | a | g | 0.5962 | -0.07 | 0.002 | 7.03E-319 | 1064.8 |  |  |  |  |  |
| 786 | rs150260898 | a | g | 0.9286 | 0.04 | 0.004 | 1.12E-25 | 95.5 |  |  |  |  |  |
| 787 | rs1001352 | t | c | 0.8165 | 0.02 | 0.002 | 2.11E-21 | 54.0 |  |  |  |  |  |
| 788 | rs2224538 | t | c | 0.6362 | 0.02 | 0.002 | 6.28E-25 | 83.4 |  |  |  |  |  |
| 789 | rs6129863 | a | g | 0.2223 | -0.02 | 0.002 | 3.10E-12 | 62.3 |  |  |  |  |  |
| 790 | rs7268343 | a | g | 0.1223 | 0.02 | 0.003 | 1.34E-09 | 38.7 |  |  |  |  |  |
| 791 | rs6063534 | t | c | 0.5187 | -0.02 | 0.002 | 1.51E-19 | 89.9 |  |  |  |  |  |
| 792 | rs2182356 | t | g | 0.7327 | -0.02 | 0.002 | 5.52E-21 | 70.6 |  |  |  |  |  |
| 793 | rs6026214 | a | c | 0.4261 | 0.01 | 0.002 | 1.34E-14 | 22.0 |  |  |  |  |  |
| 794 | rs6026578 | c | g | 0.375 | 0.02 | 0.002 | 3.08E-14 | 84.4 |  |  |  |  |  |
| 795 | rs73619441 | t | g | 0.8563 | 0.02 | 0.003 | 3.04E-09 | 44.3 |  |  |  |  |  |
| 796 | rs4818280 | t | c | 0.6269 | -0.01 | 0.002 | 2.84E-10 | 21.1 |  |  |  |  |  |
| 797 | rs73197345 | a | t | 0.1367 | 0.02 | 0.003 | 3.55E-14 | 42.5 |  |  |  |  |  |
| 798 | rs2212926 | a | c | 0.2106 | -0.02 | 0.002 | 7.75E-21 | 59.9 |  |  |  |  |  |
| 799 | rs2298333 | t | c | 0.5677 | -0.03 | 0.002 | 9.58E-44 | 199.0 | rs2230033 | rs2230033 | rs2230033 | rs2230033 | rs2230033 |
| 800 | rs8133910 | t | g | 0.256 | 0.02 | 0.002 | 3.35E-22 | 68.6 | rs2839108 | rs2839108 | rs2839108 | rs2839108 | rs2839108 |
| 801 | rs165849 | a | g | 0.6975 | 0.02 | 0.002 | 4.57E-14 | 76.0 |  |  |  |  |  |
| 802 | rs134040 | a | g | 0.5235 | 0.01 | 0.002 | 2.54E-09 | 22.5 |  |  |  |  |  |
| 803 | rs5763821 | a | c | 0.6093 | -0.02 | 0.002 | 1.06E-21 | 85.8 | rs2412970 | rs2412970 | rs2412970 | rs2412970 | rs2412970 |
| 804 | rs5750482 | t | c | 0.371 | -0.01 | 0.002 | 4.63E-12 | 21.0 |  |  |  |  |  |
| 805 | rs11570645 | a | g | 0.9424 | 0.03 | 0.004 | 1.45E-11 | 44.0 |  |  |  |  |  |
| 806 | rs2008174 | t | c | 0.7408 | 0.02 | 0.002 | 3.71E-14 | 69.2 |  |  |  |  |  |
| 807 | rs202637 | a | g | 0.1835 | -0.02 | 0.003 | 1.16E-15 | 54.0 |  |  |  |  |  |
| 808 | rs41311445 | a | c | 0.9042 | 0.03 | 0.003 | 4.73E-24 | 70.2 |  |  |  |  |  |
| 809 | rs139030 | a | g | 0.7773 | -0.02 | 0.002 | 3.07E-11 | 62.4 |  |  |  |  |  |
| 810 | rs9330813 | a | g | 0.3154 | -0.02 | 0.002 | 6.87E-15 | 77.8 |  |  |  |  |  |

ALM, appendicular lean mass; SNP, single-nucleotide polymorphism; EA, effect allele; OA, other allele; EAF, effect allele frequency; SE, standard error; F, F statistic; AIS, any ischemic stroke; LAS, large artery stroke; SVS, small vessel stroke; CES, cardioembolic stroke; AD, Alzheimer's disease.

**Supplementary Table 2.** Evaluation of heterogeneity and horizontal pleiotropy using different methods.

| Outcomes | SNPs, n | Cochran’s Q  statistic | *P*-value for Cochran’s Q | *P*-value for MR-Egger intercept |
| --- | --- | --- | --- | --- |
| AIS | 780 | 1123.318 | < 0.001 | 0.965 |
| LAS | 783 | 965.477 | < 0.001 | 0.295 |
| SVS | 778 | 979.998 | < 0.001 | 0.507 |
| CES | 778 | 923.675 | < 0.001 | 0.817 |
| AD | 786 | 963.066 | < 0.001 | 0.931 |

AIS, any ischemic stroke; LAS, large artery stroke; SVS, small vessel stroke; CES, cardioembolic stroke; AD, Alzheimer's disease; SNP, single-nucleotide polymorphism.
